# Spatialproteomics: an interoperable toolbox for analyzing highly multiplexed fluorescence image data

**DOI:** 10.1038/s41592-026-03155-1

**Published:** 2026-07-24

**Authors:** Matthias Meyer-Bender, Harald Vöhringer, Christina Schniederjohann, Sarah Koziel, Erin Chung, Ekaterina Popova, Alexander Brobeil, Nicklas Griese, Nora Kolks, Lisa-Maria Held, Aamir Munir, Scverse Community, Mikaela Koutrouli, Luca Marconato, Wouter-Michiel Vierdag, Lucas Diedrich, Vincenth Brennsteiner, Theodoros Visvikis, Jose Nimo, Panos Roussos, Erwin Schoof, Sascha Dietrich, Peter-Martin Bruch, Wolfgang Huber

**Affiliations:** 1 European Molecular Biology Laboratory (EMBL), Heidelberg, Germany; 2 Molecular Medicine Partnership Unit (MMPU), Heidelberg, Germany; 3 Department of Medicine V, Hematology, Oncology and Rheumatology, University of Heidelberg, Heidelberg, Germany; 4 Department of Hematology and Oncology, University Hospital Düsseldorf, Düsseldorf, Germany; 5 Center for Integrated Oncology Aachen–Bonn–Cologne, Düsseldorf, Germany; 6 Spatial & Functional Screening Core Facility, Medical Faculty, Heinrich Heine University Düsseldorf, Düsseldorf, Germany; 7 Department of Pathology, University of Heidelberg, Heidelberg, Germany; 8 Genentech, San Francisco, CA USA; 9 Department of Proteomics and Signal Transduction, Max Planck Institute of Biochemistry, Martinsried, Germany; 10 Maastricht MultiModal Molecular Imaging (M4I) Institute, Division of Imaging Mass Spectrometry, Maastricht University, Maastricht, Netherlands; 11 Max-Delbrück-Center for Molecular Medicine in the Helmholtz Association (MDC), Berlin, Germany; 12 Department of Medicine V, Hematology, Oncology and Rheumatology, Charité – Universitätsmedizin Berlin, Berlin, Germany; 13 Center for Disease Neurogenomics, Icahn School of Medicine at Mount Sinai, New York, NY USA; 14 Department of Psychiatry, Icahn School of Medicine at Mount Sinai, New York, NY USA; 15 Department of Genetics and Genomic Sciences, Icahn School of Medicine at Mount Sinai, New York, NY USA; 16 Center for Precision Medicine and Translational Therapeutics, James J. Peters VA Medical Center, New York, NY USA; 17 Department of Biotechnology and Biomedicine, Technical University of Denmark, Kongens Lyngby, Denmark

**Keywords:** Image processing, Software, Computational models

## Abstract

Highly multiplexed immunofluorescence imaging visualizes and quantifies protein levels at single-cell resolution in intact tissues at low cost and high scalability. Analysis of these data involves multiple steps with many method and parameter choices that must be adapted to the data and analytical objectives. There is an unmet need for a toolbox that offers flexible end-to-end coverage of the workflow. Here we present ‘spatialproteomics’, a Python package that addresses these challenges. Spatialproteomics enables the processing and analysis of large imaging data, including steps such as segmentation, image processing and cell-type classification, while synchronizing shared coordinates across data modalities. We demonstrate spatialproteomics on images of reactive lymph nodes and B cell non-Hodgkin lymphomas from 132 patients. We showcase an end-to-end analysis from raw images to statistical characterization of how cell type composition and spatial distribution vary across indolent and aggressive lymphomas. Furthermore, we show how spatialproteomics can process Gigapixel whole-slide images.

## Abstract (web summary)

Spatialproteomics is a Python-based toolbox that supports end-to-end analysis of highly multiplexed imaging data.

## Main

Recent advances in spatial proteomics, such as co-detection by indexing (CODEX)^1^, MACSima imaging cyclic staining (MICS)^2^ or imaging mass cytometry (IMC)^3^, enable highly multiplexed molecular and morphological profiling of tissues at single-cell resolution on a large scale. These techniques generate multichannel images, where each channel represents the spatial distribution of a distinct antigen. As these assays are compatible with formalin-fixed paraffin-embedded tissues, the standard-of-care for archiving diagnostic biopsies, they have found broad adaptation in oncology, where they have been used to characterize the tumor microenvironment (TME) in entities including lymphomas^4,5^, colorectal^6^ and head and neck cancers^7^.

**Fig. 1:**
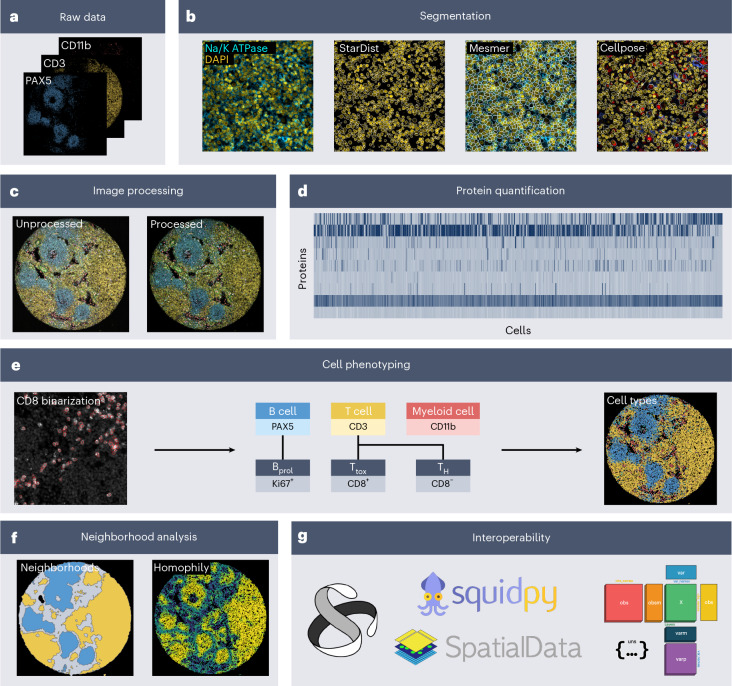
An exemplary workflow orchestrated with spatialproteomics. **a**,**b**, After raw images are obtained (**a**), segmentation is performed either on the nuclear or whole-cell level, or on multiple markers independently (**b**). **c**, Image-processing tools such as thresholding can boost the signal-to-noise ratio of the image. **d**, Fluorescence intensities are quantified for each segmented cell. **e**, The resulting expression matrix serves as input for cell type assignment, both of major cell types and subtypes. T_tox_, cytotoxic T cell. **f**, These cell type assignments can then be used to perform neighborhood analysis or find areas of tissue heterogeneity. **g**, Export into data formats like anndata and spatialdata ensures interoperability with the scverse ecosystem. Copyright © 2021, Theis Lab.

**Extended Data Fig. 1:**
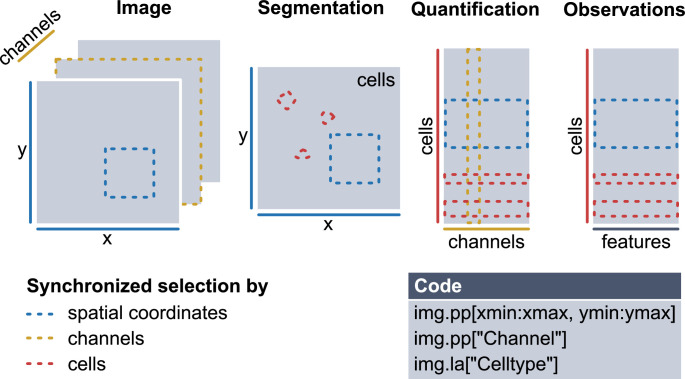
*Spatialproteomics* ensures synchronization of data with shared dimensions. Users can subselect data based on spatial coordinates, channels, cell types, and more.

Following Kuswanto et al.^8^, we here consider the following workflow consisting of cell segmentation, image processing, protein quantification and cell phenotyping (Fig. 1). This workflow requires the creation of additional data objects, such as segmentation masks and protein expression matrices. While these, as well as the primary imaging data itself, could be represented simply as a loose collection of multidimensional arrays, many of them share common dimensions (Extended Data Fig. 1). For example, the multiplexed imaging data (channels, *x*, *y*) share spatial dimensions with their corresponding segmentation masks (*x*, *y*). Similarly, the instance labels in a segmentation mask can be viewed as a dimension that aligns with the expression matrix (cells by channels) quantifying protein expression for each cell, or with a morphology matrix (cells by shape features) of shape and texture descriptors for each cell. A desirable data structure for multiplexed imaging data should keep track of such shared dimensions, ensuring that changes to one component automatically update the others to maintain consistency. For example, when subsetting a region of an image, the segmentation mask should be subset accordingly, and the expression matrix should only retain cells within this region. Similarly, it should be possible for users to select specific cell types and forward the resulting subset object to downstream methods for the computation of spatial statistics.

We introduce ‘spatialproteomics’, a Python package that leverages xarray^9^ to provide a consistent representation of multiplexed imaging data and associated data types across shared dimensions. The package offers a consistent programmatic interface for image processing, segmentation, marker quantification, cell type classification, neighborhood analysis and visualization. Functions in spatialproteomics are endomorphisms on the xarray class, which facilitates the ‘piping’ of the data through a series of such operations by function composition^10^. Furthermore, spatialproteomics can process high-dimensional larger-than-memory imaging datasets in parallel via Dask^11^, as well as integrate datasets from different tissues and using different antibody panels and methods.

Spatialproteomics interoperates with scverse, an open-source ecosystem of data structures and tools for analyzing single-cell omics data^12^, by supporting data conversion to both the anndata and spatialdata formats^13,14^. Additionally, by working directly on spatialdata objects, spatialproteomics can be incorporated into existing workflows within the scverse ecosystem. This interoperability of spatialproteomics with scverse benefits users interested in building sophisticated, multicomponent workflows, and it encourages the development of open, modular methods that can each plug into a rich environment of upstream and downstream tools. The package is available at https://github.com/sagar87/spatialproteomics.

## Results

### Spatialproteomics streamlines the processing of highly multiplexed fluorescence images

Spatialproteomics is designed for large collections of multiplexed immunohistochemistry imaging data, as can be obtained from various technology platforms. An analysis of an exemplary dataset is reported below. Generally, spatialproteomics analyses start with importing the imaging data and metadata from the instrument output, such as tagged image file format (tiff) files^15^ as well as a mapping from channels to proteins. For visualization, users can select which channels to show and assign custom colors to each channel (Fig. 1a). To facilitate interactive work and conserve memory, users can also subset or downsample images.

Functions in spatialproteomics follow an endomorphic design pattern, allowing function calls to be chained. This eliminates the need for intermediate variables or data structure conversions, ensures consistent synchronization of related data, improves code readability, and reduces memory usage, as functions return a new object that shares underlying data with the original. Because all functions in the package adhere to this pattern, complex analysis pipelines can be constructed in just a few lines of code.

A common next step is cell segmentation (Fig. 1b). Spatialproteomics offers wrappers for stardist^16^, mesmer^17^ and cellpose^18^, enabling rapid experimentation with different segmentation methods. All of these employ pretrained neural networks and provide segmentation of nuclei and optionally also of whole cells. Users can also load their own segmentation mask directly into the spatialproteomics object, making the workflow adaptable to new methods.

We have experienced incompatible dependencies between some of these tools. For example, mesmer currently only supports Python up to v.3.10, which is deprecated in newer versions of xarray. To reduce such conflicts, spatialproteomics offers multiple installation options that distinguish between mandatory and optional dependencies.

In some cases, segmentation on a nuclear or a universal membrane marker does not provide satisfactory results, and more elaborate approaches are required. For example, segmentation of macrophages or dendritic cells can be hampered by their large size and irregular shape. To counteract this problem, independent segmentations can be performed on several markers, for example, 4,6-diamidino-2-phenylindole (DAPI) (nuclei), CD68 (macrophages) and CD11c (dendritic cells), and be suitably merged.

Antibody-based imaging can suffer from high background fluorescence and high variability of binding efficiency and specificity across samples or even within the same image. To address such issues, users can apply predefined or custom-written image-processing steps, such as thresholding, filtering and morphological operations^19^ (Fig. 1c).

Next, an expression matrix is computed by summarizing intensities over each object instance (for example, cell) (Fig. 1d). Spatialproteomics offers different options, including average, sum, median, percentage of nonzero pixels; users can also provide their own summary functions. To facilitate downstream analysis and comparisons across markers and samples, transformations such as min–max normalization, *z*-score or arcsinh can be applied^20^.

Many downstream analysis approaches benefit from annotating the observed cells with cell type labels. Such cell type classification is, however, a bespoke task for which there are no general or out-of-the-box solutions. It is contingent both on the scientific goals of the study, which can direct the granularity of the cell type classification, and on technological limitations. Spatialproteomics facilitates the creation of such bespoke annotation workflows. We have successfully used a decision-tree approach (Fig. 1e). This requires the user to define a set of mutually exclusive marker proteins for each of the cell types or states they want to distinguish, analogous to gating methods used in multidimensional flow cytometry^21^. After thresholding the image for those markers to remove background signal, we assign cell types based on identifying the cell type whose corresponding marker expression is largest.

Whichever cell type annotation method users choose, we recommend designing it to be robust against so-called spillover artifacts, in which protein expression signals from a neighboring cell are counted toward the expression profile of the currently considered cell. Reasons for spillover may include segmentation errors, but also more fundamentally the fact that tissue slices have finite thickness and their two-dimensional projection may map overlapping cells onto the same pixel.

An alternative to the above supervised approach are unsupervised methods such as (spatially aware) Leiden clustering^22,23^. A potential limitation is the data-dependence of the identified clusters, which complicates comparisons between datasets or extension with additional images.

After annotation with major cell type labels, users may want to more finely phenotype the cells, for example, by considering proliferation state based upon the marker Ki-67. To enable the partitioning of major cell types into a more fine-grained classification scheme, such markers can be binarized, resulting in each cell being ‘positive’ or ‘negative’ for a given marker.

Spatialproteomics allows users to define a cell type hierarchy or gating tree, which partitions the previously predicted major cell types into more fine-grained subtypes. This scheme also enables more complex marker combinations. Next to single marker positivity or negativity, a cell subtype can also be defined by a combination of both positive and negative expression, or by a set of alternative markers (for example, a cytotoxic T cell could be labeled as ‘exhausted’ if it expresses either PD1 or TIM3).

In addition, spatialproteomics provides a wrapper around assignment of single-cell proteomics (astir)^24^, a probabilistic approach that makes cell type assignments with measures of uncertainty. For full flexibility, users can insert their own cell type labels into objects of the spatialproteomics class.

Schürch et al. introduced the concept of cellular neighborhoods to detect changes in tissue composition when comparing multiple samples^8,25–28^ (Fig. 1f). In this approach, the relative cell type abundances are computed for the neighborhood of each cell. Neighborhoods can be defined via Delaunay triangulation, the *k*-nearest neighbors or a circle with a fixed radius. The relative cell type abundance profiles are aggregated via unsupervised clustering, such as *k*-means clustering, and results may be compared across conditions. Measures such as homophily (the fraction of cells in the neighborhood with the same cell type as the center cell) and Shannon’s diversity index can be used to quantify tissue homo- or heterogeneity, and data may be subset for certain neighborhood types, for example, tumor regions or particular types of microenvironment.

Spatialproteomics provides further methods for exploring spatial and neighborhood relationships, for instance by applying graph theoretical measures on the labeled graph in which each cell is a node and edges are between cells in direct vicinity of each other.

**Extended Data Fig. 2:**
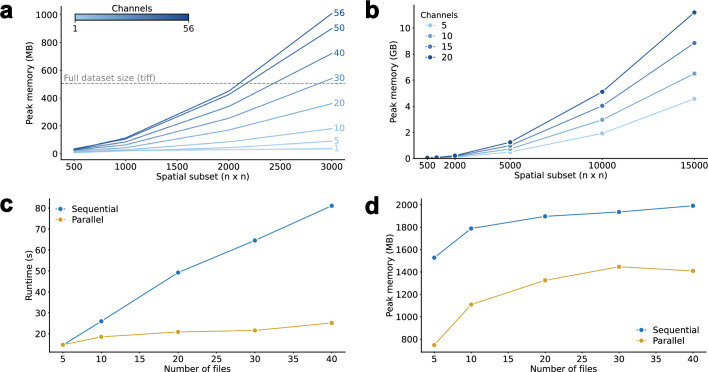
Lazy loading with *Dask* enables memory efficient subsetting of cores. **a**, Loading of different numbers of markers and spatial subsets across 40 TMA cores. When subsetting to a smaller area, or selecting a limited amount of markers, lazy loading conserves runtime compared to reading the full dataset into memory. It is however notable that when loading the full data into memory (and thereby converting the *Dask*-backed object into a numpy array), the peak memory briefly doubles. **b**, Loading of a WSI with over 100GB on disk (35,520 by 56,160 pixels, 53 channels). Subsetting by markers or a region reduces memory requirements and enables analysis on low-memory machines. **c**, Parallelization with *Dask* across 16 workers enables faster runtimes when analyzing many samples at once (here: quantification and arcsinh-transform). **d**, Parallelization across 16 workers does not come at the cost of increased memory. Instead, the peak memory per worker is reduced.

Putting everything together, spatialproteomics provides plotting methods to visualize marker intensities, segmentation masks, cell type labels and neighborhoods. On disk, objects are stored as zarr files, so that users can load image slices, segmentation masks, quantifications, cell type assignments and other possibly interesting data, without needing to bring the whole object into working memory. This design enables analysis of large datasets on compute infrastructure that would otherwise fail due to memory constraints (Extended Data Fig. 2a,b). As spatialproteomics is based on xarray, users can also make full use of Dask’s parallelization capabilities, which can reduce runtime compared to sequential processing of many samples (Extended Data Fig. 2c,d).

Customizability is a key feature of spatialproteomics. It provides wrappers for established tools and is ready to interoperate with new methods as the field evolves. Its well-documented and accessible data representation enables users to programmatically extend all aspects of functionality, for example, for segmentation, cell phenotyping, network analysis or spatial statistics.

To enable the integration with downstream analysis packages, we implemented export functions to the anndata^13^ and spatialdata^14^ formats (Fig. 1g), which can in turn be used with spatial analysis tools such as squidpy^29^, SCIMAP^28^, and Multiomics and Ecological Spatial Analysis (MESA)^30^. Spatialproteomics also supports direct manipulation of spatialdata objects, which can be ingested using tools such as spatialdata-io^14^. This interoperability also enables interactive analysis using tools such as napari^31^, enabling users to visualize, explore and manually annotate their data. Finally, we also provide guidance on how to export the data to R-based packages such as spatstat^32^ or Seurat^33^.

### Example analysis of the B cell non-Hodgkin lymphoma data

To demonstrate spatialproteomics, we considered a spatial profiling dataset of lymph node biopsies from 132 patients with B cell non-Hodgkin lymphomas (BNHL) or non-neoplastic controls. Tissue microarrays (TMAs) and whole slides were imaged with the PhenoCycler Fusion using DAPI and a set of 55 antibodies (Supplementary Table 1) selected for specific binding to proteins important in B, T and myeloid cell biology^4^. We measured 1-mm-diameter TMA cores (*n* = 250) with a median of two replicates per patient (Supplementary Table 2). Each core was cropped to an image the size of 9 megapixels (3,000 × 3,000). In addition, we generated five whole-slide images (WSIs) with 52 antibodies (Supplementary Table 3) and sizes between 0.9 and 2 gigapixels (Supplementary Table 4). We used spatialproteomics to load these data and performed an initial quality control by visually inspecting the images.

The samples were grouped into three distinct categories based on their condition: reactive lymph nodes (29 patients), indolent lymphomas with retained tissue structure (marginal zone lymphoma (MZL) and follicular lymphoma (FL), 61 patients) and aggressive lymphomas with diffuse structure (diffuse large B cell lymphoma (DLBCL), primary mediastinal large B cell lymphoma (PMBCL), Burkitt’s lymphoma and B cell lymphoblastic lymphoma (BLBL), 42 patients). In total, more than 3.5 million cells were segmented, with the average number of cells detected per core being 14,273.

**Fig. 2:**
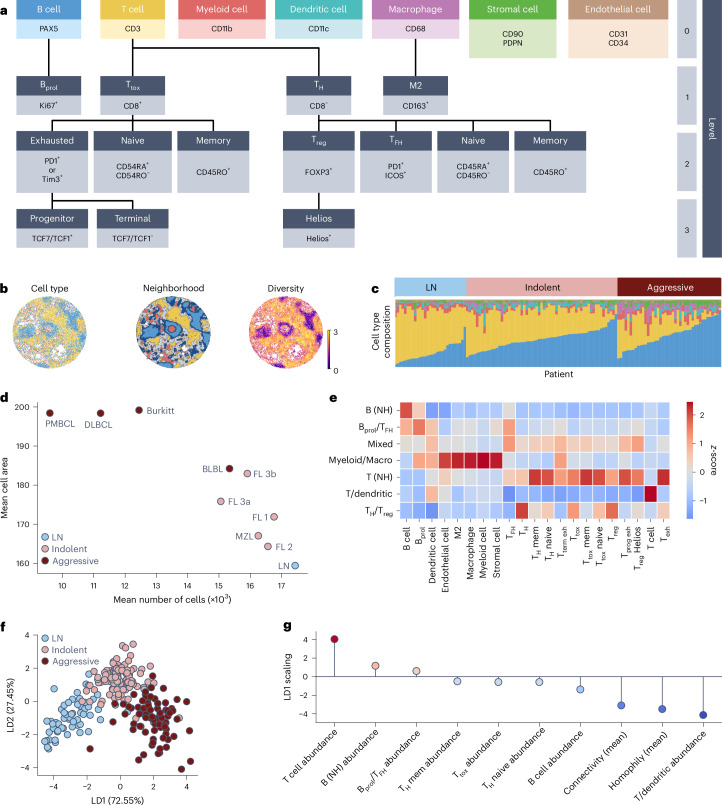
Analysis of a diverse selection of B cell non-Hodgkin lymphomas. **a**, A hierarchical gating tree used for cell type and subtype assignment. **b**, Cell types, neighborhoods and Shannon’s diversity index of a single lymph node core. Only coarse cell types are shown in the visualization. Cell type colors correspond to the ones shown in **a**. **c**, Overview of patients and their cell type compositions; colors are as in **a**. **d**, More aggressive lymphomas tend to have larger cells than lymph nodes (LNs). **e**, Clustered neighborhoods (NH) capture distinct cell type compositions. **f**, LDA of samples based on cell type abundances, neighborhood abundances, and morphological information. **g**, LD1 scalings of the individual features in the space spanned by the class centroids from the LDA. Only features with an absolute scaling above 0.5 are shown. Colors correspond to the LD1 scaling shown on the *y* axis.

BNHL consist of malignant B cells along with various other cell types in the TME. Although the composition of the TME is heterogeneous across disease subtypes, several major cell types, such as T cells, macrophages and endothelial cells, are commonly present^34^. These can be subdivided further according to the expression of additional lineage-specific or functional markers. Previous studies showed that the frequencies of specific T cell subpopulations vary across BNHL subtypes, partly due to differences in clonal expansion, and can be associated with survival^4^. To explore this, we constructed a hierarchical gating tree that focused specifically on the classification of T cell subsets (Fig. 2a).

**Extended Data Fig. 3:**
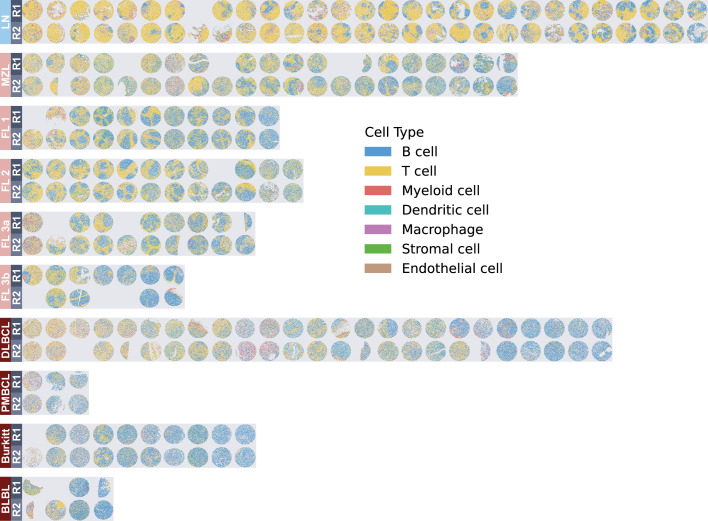
Coarse cell type assignments for all samples, stratified by entity. Replicates are shown above one another. *n* = 57 LN, 39 MZL, 21 FL 1, 23 FL 2, 18 FL 3a, 11 FL 3b, 49 DLBCL, 6 PMBCL, 19 Burkitt, 7 BLBL.

**Extended Data Fig. 4:**
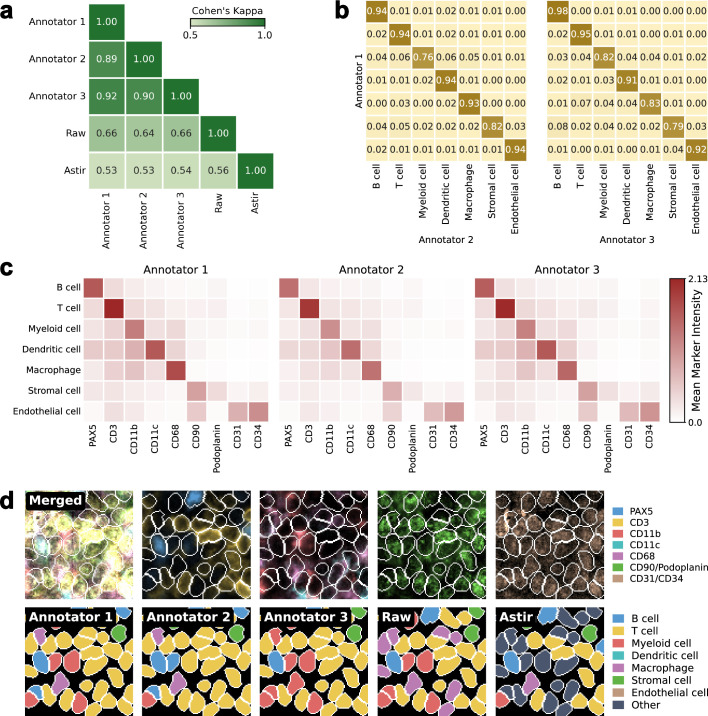
Manual thresholding was robust across independent annotators and entities. **a**, Cohen’s Kappa between three independent human annotators, assignments on the unthresholded images, and astir. Human annotators agreed more with one another than with the fully automated approaches. **b**, Confusion matrices show high agreement between annotators. **c**, Mean marker intensities for each cell type after thresholding. Despite the thresholding being performed independently, the resulting marker profiles looked similar across annotators. **d**, Exemplary cell type assignments and the corresponding markers. The top row also shows the disentangled marker intensities for better visibility.

We manually thresholded each marker and annotated the cell type of each individual cell according to this gating tree (Fig. 2b and Extended Data Fig. 3). As our cell type assignment was preceded by a manual selection of thresholds, we aimed to control inter-annotator variability. To this end, we selected 40 cores (20 FL and 20 DLBCL) and asked three different human annotators to perform the threshold selection independently. We also considered two alternative classification methods: assigning cell types based upon which marker had the highest mean expression in a cell without any thresholding and astir, a fully automatic method^24^. The classifications from the three annotators agreed more with one another than with the assignment from the raw intensities or astir (Extended Data Fig. 4a,b). The average thresholded marker profiles per cell type looked very similar across annotators (Extended Data Fig. 4c). A major source of discrepancy between the human annotations and astir was the fact that astir was given the option of assigning the cell type ‘Other’ for 16.7% of all cells, whereas the human-thresholded gating tree always assigns one of the predefined cell types (Extended Data Fig. 4d).

**Extended Data Fig. 5:**
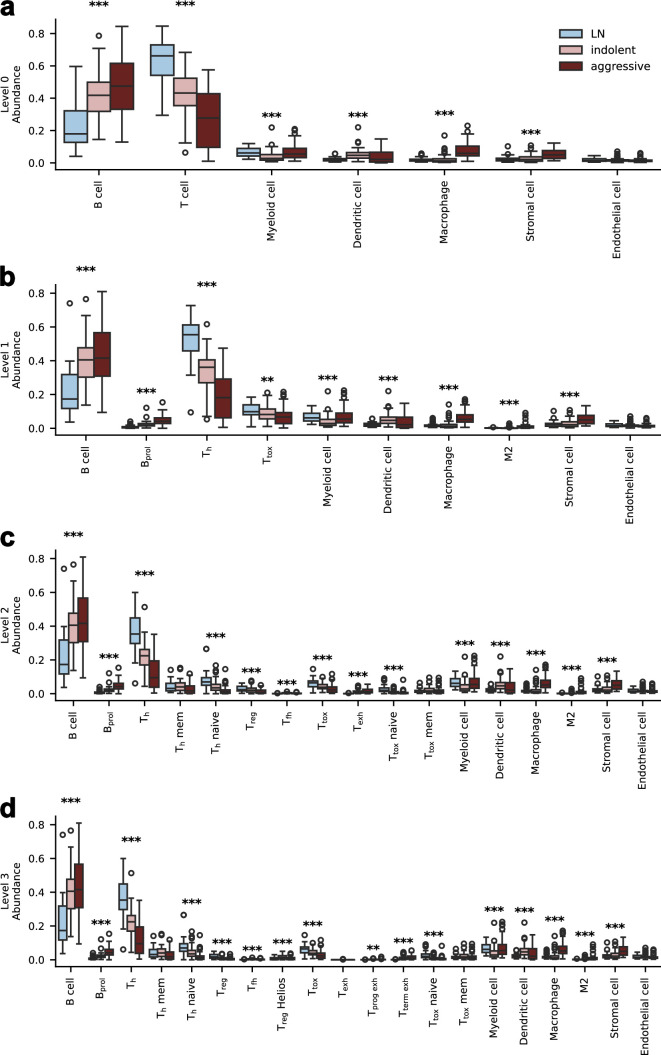
Cell type abundances quantified at different granularities of the gating tree. Box plots show the median (center line), interquartile range (box), and whiskers extending to 1.5 times the interquartile range. Statistical significance was assessed using ANOVA. Significance levels are indicated as follows: p < 0.05 (*), p < 0.01 (**), and p < 0.001 (***). *n* = 29 LN, 61 indolent, 42 aggressive. **a**, Abundances at level 0. **b**, Abundances at level 1. **c**, Abundances at level 2. **d**, Abundances at level 3. Source data contain exact *P* values. Source data

**Extended Data Fig. 6:**
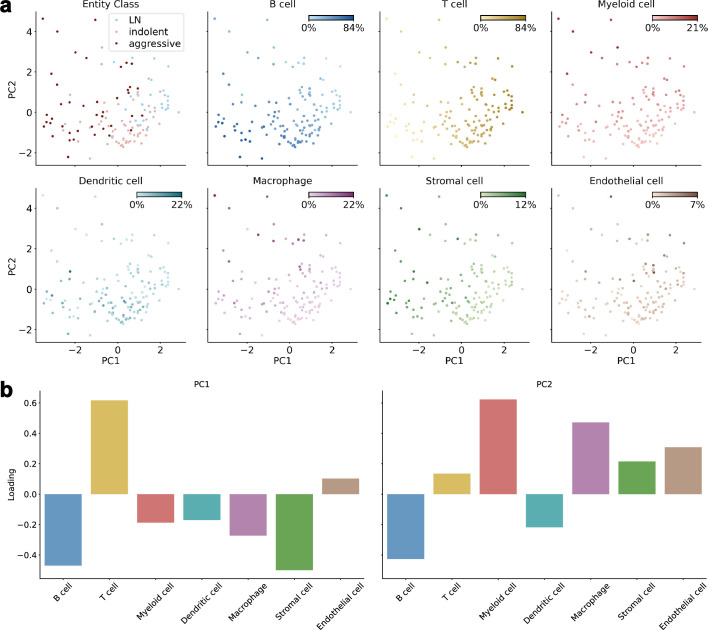
PCA of the patients in the BNHL cohort computed on the relative cell type abundances. **a**, PC1 vs PC2, colored by entity class and abundance of the major cell types. **b**, Loadings of the first two principal components show that PC1 is dominated by differences in B, T, and stromal cell composition. PC2 distinguishes based on B, myeloid, endothelial cells and macrophages.

A comparison of cell type composition showed that lymphoma samples had a substantially higher content of B cells (Fig. 2c and Extended Data Fig. 5), which is expected due to accumulation of malignant B cells. Furthermore, we noted an increase in macrophages and stromal cells pointing toward increased vascularization of the tumor samples (Extended Data Fig. 5). A principal-component analysis based on relative cell type abundances corroborated this finding, showing that the first axis of variation was driven by B, T and stromal cell abundances, whereas the second one reflected differences in macrophage and myeloid cell abundances (Extended Data Fig. 6).

In addition to cell type composition, we investigated cell areas in the different conditions. Aggressive lymphomas showed larger cells, while the average cell area in indolent lymphomas was closer to that in reactive lymph nodes (Fig. 2d), in line with the blast-like morphology of aggressive lymphoma^35^. As a consequence, cores from aggressive lymphomas contained fewer cells in the same tissue area.

**Extended Data Fig. 7:**
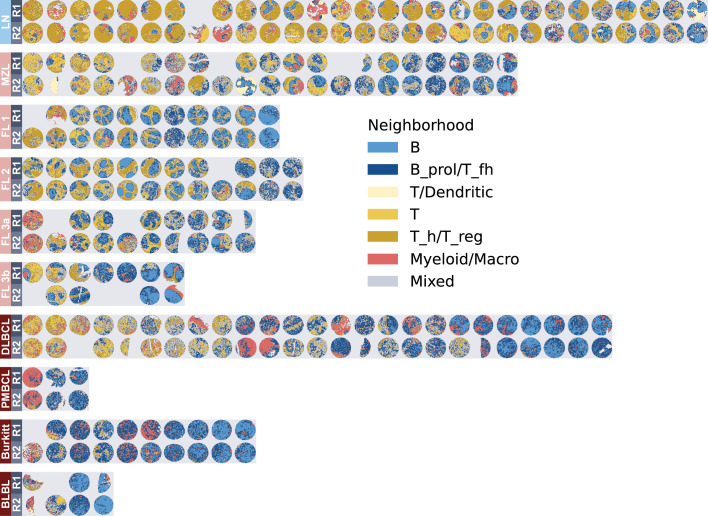
Neighborhood assignments for all samples, stratified by entity. Replicates are shown above one another. *n* = 57 LN, 39 MZL, 21 FL 1, 23 FL 2, 18 FL 3a, 11 FL 3b, 49 DLBCL, 6 PMBCL, 19 Burkitt, 7 BLBL.

To further characterize tissue architecture, we computed the cell type composition within a radius of 25 μm around each cell and clustered these composition profiles across samples to obtain typical neighborhood patterns (Fig. 2e). We identified seven distinct neighborhoods, including a B cell-rich neighborhood (B (NH)) and one enriched in proliferating B (B_prol_)/follicular helper T (T_FH_) cells. Another neighborhood consisted mainly of myeloid cells and macrophages (Myeloid/Macro) and one showed enrichment in helper T (T_H_) and regulatory T (T_reg_) cells. The ‘T/dendritic’ neighborhood predominantly captured T cells that could not be further classified due to limited CD8 signal. While this reflects a limitation of the hierarchical cell type classification in low signal-to-noise settings, we observed this neighborhood in only a few samples (Extended Data Fig. 7).

**Extended Data Fig. 8:**
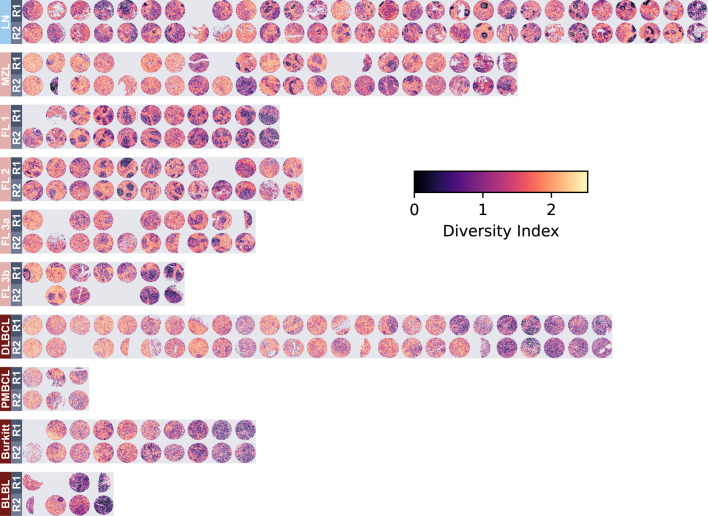
Shannon’s diversity index of a cell’s microenvironment. Due to T cells being classified at higher granularity, T cell-rich zones tend to look more heterogeneous than B cell-rich zones. *n* = 57 LN, 39 MZL, 21 FL 1, 23 FL 2, 18 FL 3a, 11 FL 3b, 49 DLBCL, 6 PMBCL, 19 Burkitt, 7 BLBL.

Given cell type abundances, neighborhood abundances, as well as morphological information such as the mean homophily or Shannon’s diversity index (Extended Data Fig. 8) of every patient, we were interested to see which of these metrics could be used to differentiate between lymph nodes, indolent and aggressive lymphomas. We performed a linear discriminant analysis on the three groups, which showed a clear trajectory from reactive lymph nodes over indolent to aggressive lymphomas (Fig. 2f). To see which features were most important for the classification, we considered the scaling of each feature in the space spanned by the class centroids (Fig. 2g). Aside from cell type abundances, one major driving factor was homophily, which is in line with observed diffuse architecture in most aggressive lymphomas, showing that there is merit in obtaining descriptors beyond cell type abundances.

#### Spatial statistics reveal spatial associations between cell types

**Fig. 3:**
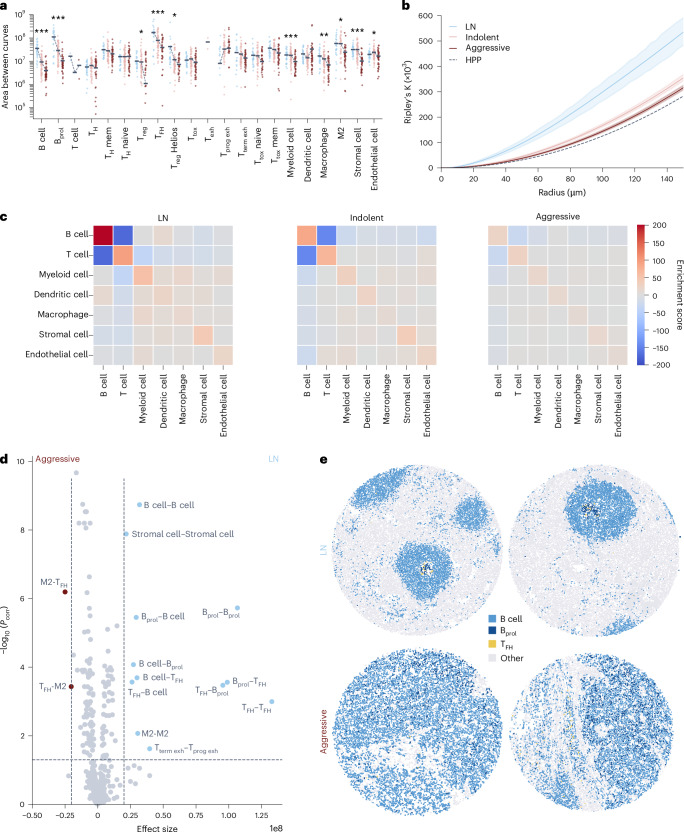
Spatial analysis of the BNHL cohort. **a**, Area between theoretical and observed Ripley’s K function. Several cell types (B cells, B_prol_, T_FH_…) cluster more in LNs and lose structure in lymphomas. Lines indicate the mean; *n* = 29 LNs, 61 Indolent, 41 Aggressive; colors as in **b**. Statistical significance was assessed by analysis of variance (ANOVA). Significance levels are indicated as follows: *P < 0.05, **P < 0.01, ***P < 0.001. Exact *P* values are available in the Source Data. **b**, Ripley’s K for B cells, resolved by entity class. The black line shows the expected distribution if the point distribution followed a homogeneous Poisson process (HPP). Data are shown as mean ± 95% CI. **c**, Mean neighborhood enrichment *z*-scores from squidpy on level 0 of the gating tree. Spatial associations diminish in more aggressive lymphomas. **d**, Ripley’s Cross-K function on the fine-grained cell types. Differences between LN and aggressive, *P* values determined with an independent two-sided *t*-test. **e**, Representative cores for LN and aggressive lymphomas show that spatial organization gets lost. Source data contain exact *P* values from **a**. Source data

While cellular neighborhoods provide one perspective into the spatial distribution of cell types, there are many more spatial statistics methods to further investigate and characterize tissue architectures, cell-to-cell interactions, and higher-order patterns. We set out to identify which cell types occurred more frequently in clusters and which ones showed a more random spatial distribution. We computed Ripley’s K function up to a distance of 150 μm and looked at the area between the observed curve and the null curve of a homogeneous Poisson process with the same intensity. Figure 3a,b shows that B cells, B_prol_ cells and T_FH_ cells occurred in spatial clusters in reactive lymph nodes, corresponding to germinal centers. The clustering was less pronounced in aggressive lymphomas, consistent with their more dispersed tissue structure.

We asked whether there were certain combinations of cell types that frequently colocate by computing squidpy’s neighborhood enrichment score^29^ at distances of <70 μm. We applied this to the coarse cell types, which showed that the structure becomes more diffuse with the aggressiveness of the lymphomas (Fig. 3c).

Next, we extended this analysis to the fine-grained cell type annotations. We computed the areas between the observed and theoretical Ripley’s Cross-K function for each pair of cell types, and compared them between lymph nodes and aggressive lymphomas (Fig. 3d). As before, the majority of significant differences were spatial associations that were present in lymph nodes and disappeared in the lymphoma samples. While most of these associations were between cells of the same cell types, some associations reminiscent of germinal centers were also observed, such as between proliferating B cells and T_FH_ cells (Fig. 3e). On the flip side, a spatial association between M2 macrophages and T_FH_ cells seemed to be enriched in aggressive lymphomas.

#### Validation using five DLBCL whole-slide images

TMA cores only show a small part of the diagnostic biopsy, which again is just a subset of the tumor manifestation. It is a recurrent question to ask how representative a core is of the whole tissue. We performed whole-slide imaging on five samples from patients with DLBCL, for which we had two matched TMA cores each. After segmentation with cellpose and the removal of cells which were not part of the main tissue of interest, we obtained a dataset of over 6.6 million cells, which is almost twice as much as all the previous TMAs together.

**Fig. 4:**
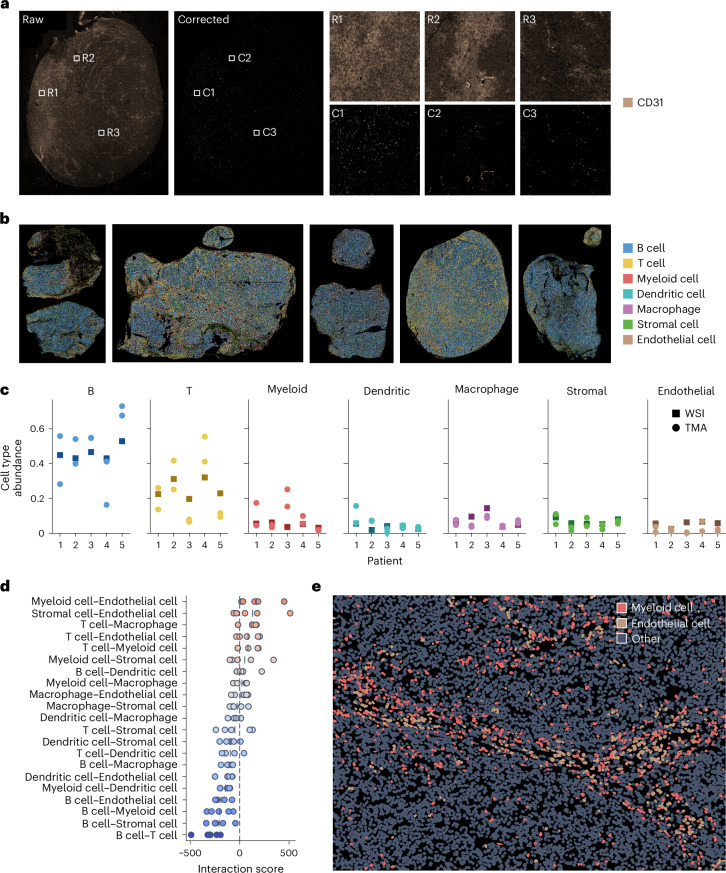
Analysis of five DLBCL whole-slide images. **a**, Spatialproteomics enables the application of arbitrary image-processing algorithms to multichannel images, allowing us to correct for differences in marker intensities across WSIs. **b**, Assigned cell types. **c**, Comparison of cell type abundances between whole-slide images and corresponding TMA cores. **d**, Neighborhood enrichment scores from squidpy, excluding self-associations. Sorted by mean enrichment score, which is indicated both in color and as vertical lines. **e**, Example of a spatial association between myeloid cells and endothelial cells.

Despite Akoya’s built-in illumination correction using the BaSiC algorithm^36^, we still observed differences in marker intensities across tissues, hampering the application of our thresholding approach. To adjust for these, we leveraged spatialproteomics’ ability to apply arbitrary image-processing methods to the multichannel images. For each channel, we constructed a second image that was a Gaussian blur of the original image, with a kernel size of 51 pixels, and subtracted it from the original one (Fig. 4a). This reduced the image-wide gradients, making it possible to apply thresholding again and use the processed images to assign cell types as previously described (Fig. 4b).

Next, we were interested in comparing cell type abundances between the WSIs and their corresponding TMA samples (Fig. 4c). While the general trend in cell type composition with many B and T cells held true in the whole-slide dataset, there were some discrepancies to the TMA results. For example, the WSI for patient 2 contained some connective tissue, which was mostly classified as myeloid cells and therefore skewed the ratio of cell types.

To make use of the spatial context, we again applied the neighborhood enrichment method from squidpy (Fig. 4d). For clarity, we only considered spatial associations between different cell types, as self-associations were frequently the most enriched. In accordance with our TMA results, B cells and T cells had a negative neighborhood enrichment score. We also found several enriched spatial associations such as myeloid–endothelial, stromal–endothelial and T–macrophage (Fig. 4e). While these were computed without taking into account a negative control, they do show that there are spatial associations between specific cell types in DLBCL, providing opportunities for future research.

## Discussion

The development of spatialproteomics has drawn inspiration and used experience from existing approaches (Table 1). In the R ecosystem, cytomapper and imcRtools^37^ build upon the SpatialExperiment data structure^38^ for spatial single-cell data. In Python, Spatial Omics Pipeline and Analysis (Sopa)^39^ and Structured Spatial Analysis for Codex Exploration (SPACEc)^40^ address the processing and analysis of highly multiplexed fluorescence images. Additionally, pipelines such as Multiple Choice MICROscopy (MCMICRO)^41^ and Single-cell Identification from MultiPLexed Images (SIMPLI)^42^ provide Nextflow-based solutions.

**Table 1:** Comparison of different tools for analyzing highly multiplexed immunofluorescence images

Package	spatialproteomics	sopa	SPACEc	MCMICRO	SIMPLI	imcRtools steinbock
**Language**	Python	Python, Snakemake	Python	Nextflow	Nextflow	R, Python
**Data format**	Xarray or spatialdata	Spatialdata	Tif, csv, …	Tif, csv, …	Tif, csv, …	SpatialExperiment
**Synchronization of shared coordinates**	Yes	No	No	No	No	Yes
**Segmentation**	Cellpose, Mesmer, Stardist	Cellpose	Cellpose, Mesmer	UnMICST, S3segmenter	CellProfiler, Stardist	Cellpose, Mesmer, CellProfiler
**Image processing**	Fully customizable	None	None	None	Thresholding	Hot pixel removal
**Protein quantification**	Fully customizable	Mean, min, max	Mean	MCQuant	CellProfiler	Mean
**Expression matrix processing**	*z*-score, Double *z*-score, MinMax, ArcSin, Clip	*z*-score, Spillover Correction	*z*-score, Double *z*-score, MinMax, ArcSin	Log1p, Rescale, ComBat	CellProfiler	Spillover compensation
**Cell phenotyping**	Astir, Gating (Argmax, Binarization)	Argmax	SVM, STELLAR, Clustering	SCIMAP (clustering, gating)	Clustering, thresholding	Random forest, clustering
**Neighborhood analysis**	Clustering	NA	Clustering	SCIMAP (Spatial LDA, Spatial Lag)	Clustering	Clustering, lisaClust
**Interactivity**	Napari	Napari	TissUUmaps	Napari (CyLinter), Minerva	NA	Napari (napari-imc), cytoviewer/ cytomapper

For the analysis workflow proposed here, we have opted to employ manual thresholding of markers for cell type assignment. While fully automated methods like astir^24^ exist, they may be sensitive to variable data quality or batch effects, which means in practice that satisfactory results will still require manual intervention, for quality assessment of input data and of output results. Thus, while we anticipate better automation resulting from advances in assay standardization or computational reasoning, the current human-in-the-loop approach is intended to make best use of the data.

A recurrent study design pattern is the use of multiple spatial profiling platforms, or of the same platform but with different antibody panels, on the same samples. For such studies, we recommend as a relatively broadly applicable and generic approach to perform separate analyses per platform, resulting in cell coordinates, cell type assignments and optionally, further markers of cell state computed from specific protein levels. The resulting labeled point processes can then be compared and integrated using spatial statistics methods^43^. For specific study designs and analytical questions, it may be possible to perform data integration already upstream, for example, by mapping marker sets into a common latent space^44,45^.

As spatial omics technologies and analysis techniques continue to develop, spatialproteomics is designed to evolve alongside. While the package’s open and modular design naturally allows incorporation of external tools into a workflow, we intend to improve usability and robustness by further work on integration or wrapping of such tools with a consistent programmatic interface.

Through its interoperability with the spatialdata format for data representation, spatialproteomics supports the combined analysis of spatial proteomics and spatial transcriptomics data within a unified data structure, thereby opening new avenues for multimodal investigations.

## Methods

### Sample preparation

Microscopy slides with 5-μm sections of the BNHL TMAs and DLBCL WSIs were prepared by the National Center for Tumor Diseases tissue bank in Heidelberg and approved by the ethics committee of the Medical Faculty Heidelberg (ethics vote S-686/2018). Multiplex immunofluorescence was performed as previously described^46^. In brief, the tissue samples were deparaffinized and re-hydrated. Heat-induced antigen retrieval was performed for 20 min at 155–160 ^∘^C using Tris–EDTA buffer at pH 9. To reduce autofluorescence, tissue samples were bleached two times for 45 min using H_2_O_2_. Tissue samples were incubated with a cocktail of 55 (52 for the WSIs) DNA oligonucleotide-conjugated antibodies overnight at 4^ ∘^C (Supplementary Tables 1 and 3). For antibody conjugation, DNA oligonucleotide sequences previously published were used^25^. The next day, a three-step fixation followed, including incubation with 1.6% paraformaldehyde (PFA), 100% methanol and bissulfosuccinimidyl suberate (BS3).

Tissue samples were imaged using the Phenocycler Fusion System (Akoya Biosciences) according to the manufacturer’s instructions. For cyclic imaging, Atto550 and Atto647N labeled complementary DNA barcodes were used. Acquired raw files were processed into a single qpTIFF file by the Fusion software (v.1.0.7 for TMAs and v.2.2.0 for WSI). Details on raw data processing are available in the manufacturer’s technical documentation (https://help.codex.bio/codex/processor/technical-notes). In brief, for each cycle, the nuclear stain pattern was compared to the reference cycle (by default, the DAPI signal from the second cycle). The calculated offsets were used to align all cycles through rigid translation.

Background subtraction was performed using ‘blank’ cycles at the beginning and end of each experiment. Deconvolution was carried out using the Microvolution package, while shading correction was applied with BaSiC. Image registration of adjacent tiles was performed using the Microscopy Image Stitching Tool library.

### TMA cropping

A grid defining the locations of the cores within the tissue microarrays was manually constructed in QuPath (v.0.4.3) using the built-in TMA de-arrayer. The coordinates were exported to a csv, and crops of size 3,000 by 3,000 pixels were extracted around the centroid of each crop.

### Segmentation

For the TMAs, cells were segmented on the DAPI channel using the cyto3 model from cellpose v.3.0.7 and filtered to exclude cells with an area below 50 pixels or above 300 pixels. Subsequently, masks were expanded into each direction by two pixels.

### Quality control

We manually excluded 28 samples from the original dataset due to artifacts such as tissue dissociation, marker spillage, or other contamination of the image. We also only kept samples for which clinical annotations were available. For samples where only part of it was corrupted by an artifact, we manually drew in masks and removed all cells within those masks from downstream analysis.

On the cell level, cells which were too isolated from other cells were removed. We achieved this by running a binary dilation of 25 pixels on the segmentation masks, finding connected components and removing components which contained less than five cells.

### Image processing and cell phenotyping

For the classification of major cell types, we first thresholded the different markers by quantiles, depending on the specificity and overall signal intensity of each marker. We started with the following default values: PAX5, 0.8; CD3, 0.7; CD11b, 0.8; CD11c, 0.9; CD68, 0.9; CD31, 0.95; CD34, 0.95; CD90, 0.95; and Podoplanin, 0.95. We then manually cross-checked the images with the cell type assignments and adjusted the thresholds where we deemed necessary. The thresholding itself consisted of computing the corresponding quantile value, subtracting it from the original image and then clipping the result to 0 to remove negative values.

After thresholding on the pixel level, we aggregated the marker intensities by taking the mean over each segmentation mask. We then applied an arcsinh transform with a cofactor of 5 to the resulting expression matrix. Finally, we assigned the cell type whose corresponding marker had the largest expression. The correspondence between cell types and markers is shown in Fig. 2a (level 0).

For the functional markers, we once again thresholded the markers manually, and then computed the percentage of positive pixels which remained within each segmentation mask after thresholding. Using a second threshold *t*, cells with *t*% or more positive pixels were assigned as ‘positive’, while all others were considered ‘negative’.

In some samples, certain markers were too unspecific to enable reliable thresholding. In such cases, we stopped classification at the corresponding node of the gating tree (Fig. 2a). For example, when we were not able to binarize CD8, we simply called all T cells ‘T cells’, and did not traverse the gating tree further. The advantage of this was that we could retain samples even if an individual marker did not exhibit specific staining characteristics.

To assess the robustness of the thresholding-based cell type assignment (Extended Data Fig. 4), we had 40 samples (20 FL and 20 DLBCL) annotated by three independent reviewers, starting from the same default values for each marker.

### Neighborhood analysis

To define neighborhoods, we considered cells whose centroids were within 25 μm from one another. After quantifying the cell type abundances within the 25 μm radius around each cell, we applied *k*-means clustering with *k* = 7 to obtain neighborhood labels. The numerical labels were then manually substituted by more informative names based on the composition of each cluster (Fig. 2e).

### Morphological profiling of niches

We implemented various metrics to quantify the heterogeneity of the environment around each cell. These metrics include:

1. Degree: the number of cells connected to each cell.
2. Homophily: the fraction of neighboring cells which have the same cell type as the center cell.
3. Inter-label connectivity: the fraction of neighboring cells which have a different cell type than the center cell.
4. Shannon’s diversity index: $H{\prime} =-\mathop{\sum }\nolimits_{i = 1}^{S}{p}_{i}\,\ln ({p}_{i})$ where *p*_*i*_ are the cell type proportions.

To compare these between samples, we computed the mean across all cells within a sample.

### Spatial statistics

To investigate the clustering behavior of individual cell types, we used the spatstat package (v.3.1) in R to compute Ripley’s K function in each sample on each cell type. We then limited the distance to 150 μm and computed the area between the observed distribution and the theoretical distribution that would emerge if the point pattern was generated by a homogeneous Poisson process.

To show the average lines in a single plot (Fig. 3b), we interpolated the data along 100 points between the distances of 0 and 150 μm using one-dimensional linear interpolation.

For squidpy (v.1.6.2), we used a radius-based definition of neighborhoods, defining two cells to be spatial neighbors if their centroids were less than 70 μm apart.

To further investigate spatial associations between cell types, we also computed Ripley’s Cross-K function using the spatstat package. We then computed the area between the observed and theoretical curves between 0 and 150 μm, and performed an independent *t*-test to evaluate the differences between lymph nodes and aggressive lymphomas (Fig. 3d). We used Benjamini–Hochberg correction with a family-wise error rate of 0.05 to adjust the *P* values. Spatial associations were deemed significant if their false discovery rate (FDR)-corrected *P* value was below 0.05 and the effect size was above 20 million (Fig. 3d).

### Analysis of whole-slide images

We segmented the WSIs using the cyto3 model from cellpose v.3.1.0 and removed cells whose area was outside of the range between 50 and 600 pixels. In addition, we drew regions of interest around the main tissue to remove outlying or irrelevant cells.

To avoid marker gradients skewing the cell type assignment, we applied a background correction by creating a copy of the original image, applying a Gaussian blur with a kernel size of 51 pixels and subtracting the result from the original image. Cell type assignment was performed as before by setting a threshold for each marker and assigning the cell type with the highest expression per cell.

### Reporting summary

Further information on research design is available in the Nature Portfolio Reporting Summary linked to this article.

## Online content

Any methods, additional references, Nature Portfolio reporting summaries, source data, extended data, supplementary information, acknowledgements, peer review information; details of author contributions and competing interests; and statements of data and code availability are available at 10.1038/s41592-026-03155-1.

## Supplementary information

Supplementary Information Supplementary Tables 1–4.

Reporting Summary

Peer Review File

## Source data

Source Data Fig. 3 and Extended Data Fig. 5 *P* values from ANOVA.

## Data Availability

Raw images and all derived data are available in the BioStudies database (https://www.ebi.ac.uk/biostudies/bioimages/studies/S-BIAD2100) under accession number S-BIAD2100 (ref. ^47^). Source data are provided with this paper.

## Code availability

Spatialproteomics can be installed via pip, and the code is publicly available on GitHub at https://github.com/sagar87/spatialproteomics under the MIT license. Documentation can be found under https://sagar87.github.io/spatialproteomics. Code to reproduce the figures shown in this paper can be found on GitHub at https://github.com/MeyerBender/spatialproteomics_bnhl.
