# Supplementary material for: Spatialproteomics: an interoperable toolbox for analyzing highly multiplexed fluorescence image data
Source: Nat Methods. 2026 Jul 24;23(8):1564–73. doi: 10.1038/s41592-026-03155-1 (PMC13441886; doi:10.1038/s41592-026-03155-1)
Supplement: Supplementary file 1 [file 41592_2026_3155_MOESM1_ESM.pdf]

nature methods

Supplementary information https://doi.org/10.1038/s41592-026-03155-1

Spatialproteomics: an interoperable toolbox for analyzing highly multiplexed fluorescence image data

In the format provided by the authors and unedited

Supplementary Information

Supplementary Tables.. 1

Supplementary Tables

Cycle	Target	Clone	Manufacturer	Catalog	Oligo	Fluorophore	Dilution (1:x)	Exposure time (ms)
1	IKZF2 (Helios)	22F6	Biolegend	137202	7	ATTO550	100	600
1	CD10	E5P7S	CST	#54370	3	ATTO647	100	150
2	TCF7/TCF1	Polyclonal	R&D	AF5596	5	ATTO550	600	300
2	PD-L1	E1L3N	Cell Signaling Technology	85164	25	ATTO647	150	250
3	BCL-6	K112-91	BD	561520	58	ATTO550	200	300
3	FoxP3	236A/E7	Invitrogen	14-4777-82	61	ATTO647	2400	300
4	CD69	poly (AF2359)	Novus	AF2359	36	ATTO550	150	300
4	Perforin	B-D48	Abcam	ab47225	51	ATTO647	300	150
5	CD19	LE-CD19	Bio-RAD	MCA2454	60	ATTO550	150	150
5	LAG3	D2G4O	CST	25848SF	42	ATTO647	600	200
6	CD21	Bu32	Biolegend	354902	21	ATTO550	200	600
6	CD62L	B-8	Santa Cruz	sc-390756	57	ATTO647	800	300
7	c-myc	9.00E+10	R&D	MAB3696	53	ATTO550	300	150
7	CD138	B-A38	Novus	NBP3-14568	76	ATTO647	400	300
8	CD15	MMA	BD	559045	14	ATTO550	10000	100
8	BCL-2	C-2	Santa Cruz	sc-7382	69	ATTO647	500	150
9	CD56	MRQ-42	Cell Marque	custom	29	ATTO550	100	400
9	IKZF3	OTI7E11	OriGene	CF804396	15	ATTO647	1600	200
10	CD25	4C9	Cell Marque	custom	24	ATTO550	300	250
10	NOXA	114C307	Abcam	ab13654	72	ATTO647	800	200
11	Tim3	Polyclonal	R&D	AF2365	44	ATTO550	150	400
11	Serpin B9	7D8	Santa Cruz	sc-59983	43	ATTO647	400	300
12	Podoplanin	D2-40	Biolegend	916606	32	ATTO550	400	500
12	CD38	EPR4106	AbCam	ab176886	66	ATTO647	150	300
13	SPARC	AON-5031	Santa Cruz	sc-73472	75	ATTO550	400	300
13	ICOS	D1K2T	CST	39740	74	ATTO647	600	300
14	CXCR5	D6L3C	Cell Signaling	custom	62	ATTO550	400	500
14	CD163	EDHu-1	Novus	NB110-40686	45	ATTO647	800	100
15	FADD	EPR5030	Abcam	ab229444	71	ATTO550	400	300
15	p53	DO-7	Santa Cruz Biotechnology	sc-47698	52	ATTO647	3200	300
16	Collagen IV	EPR20966	Abcam	ab226485	38	ATTO550	300	600
16	CD4	EPR6855	Abcam	ab181724	20	ATTO647	2400	150

17	CD7	MRQ-56	Cell Marque	custom	80	ATTO550	500	400
17	Kappa	L1C1	Cell Marque	short	70	ATTO647	1800	150
18	CD20	rIGEL/773	Novus	NBP2-54591	48	ATTO550	2400	150
18	CD34	QBEnd/10 + HPCA1/763	Novus	NBP2-47909	33	ATTO647	400	100
19	PAX5	D7H5X	CST	93009SF	11	ATTO550	1600	100
19	PD-1	D4W2J	CST	63815SF	23	ATTO647	800	250
20	CD45RA	HI100	Biolegend	304102	63	ATTO550	1600	100
20	CD11b	EPR1344	Abcam	ab216445	28	ATTO647	3200	200
21	Lambda	Lamb14	Cell Marque	short	26	ATTO550	1600	300
21	CD57	HCD57	MyBioSource	MBS140192	30	ATTO647	1200	100
22	CD11c	EP1347Y	AbCam	ab216655	49	ATTO550	800	300
22	CD90	EPR3132	Abcam	ab181885	59	ATTO647	3200	150
23	HLA DR	EPR3692	Abcam	ab215985	65	ATTO550	2400	100
23	CD68	KP-1	Biolegend	916104	41	ATTO647	4800	150
24	CD31	C31.3 + C31.7 + C31.10	Novus Bio	NBP2-47785	68	ATTO550	800	300
24	CD45	2B11 + PD7/26	Novus	NBP2-34528	56	ATTO647	900	150
25	CD3	MRQ-39	Cell Marque	short	77	ATTO550	2400	150
25	Cytokeratin	C11	Biolegend	628602	67	ATTO647	3200	80
26	CD45RO	UCH-L1	NovusBio	NBP2-33104	79	ATTO647	200	300
27	CD8	C8/144B	NovusBio	NBP2-34588-0.1mg	8	ATTO647	3600	100
28	Granzyme B	EPR20129-217	Abcam	ab219803	81	ATTO647	3600	75
29	CD79a	JBC117	Cell Marque	custom	46	ATTO647	2400	150
30	Ki-67	B56	BD	556003	6	ATTO647	3200	50

Supplementary Table 1: Overview of the antibody panel for the TMAs.

entity	entity_class	n_samples	n_female	n_male	age_min	age_median	age_max	num_cells_min	num_cells_median	num_cells_max
LN	LN	57	30	27	42	66	83	8801	17532	23330
FL 1	indolent	21	15	6	47	64	79	7936	15741	23428
FL 2	indolent	23	10	11	49	58	74	10946	16241	22314
FL 3a	indolent	18	5	11	28	52	80	4261	14195	20092
FL 3b	indolent	11	5	6	28	50	92	12432	14977	19627
MZL	indolent	39	11	26	38	64	83	6716	15942	20932
DLBCL	aggressive	49	19	30	33	75	86	3001	10368	17170
Burkitt	aggressive	19	5	14	8	26	75	3157	12462	15234
PMBCL	aggressive	6	2	2	27	38	50	7620	8796	12140
BLBL	aggressive	7	0	7	18	49	54	5379	13365	23411

Supplementary Table 2: Overview of the TMA dataset.

Cycle	Target	Clone	Manufacturer	Catalog	Oligo	Fluorophore	Dilution (1:x)	Exposure time (ms)
1	Tim3	Polyclonal	R&D	AF2365	44	ATTO550	150	400
1	CCR7	2H4	BD	550937	6	ATTO647	300	450
2	BCL-6	K112-91	BD	561520	58	ATTO550	150	600
2	CD38	E7Z8C	Cell Signaling Technology	43382SF	57	ATTO647	3000	250
3	IKZF2 (Helios)	22F6	Biolegend	137202	7	ATTO550	75	800
3	PD-L1	E1L3N	Cell Signaling Technology	85164	25	ATTO647	150	250
4	CD69	poly (AF2359)	Novus	AF2359	36	ATTO550	150	500
4	IKZF3	OTI7E11	OriGene	CF804396	15	ATTO647	1600	200
5	c-myc	9.00E+10	R&D	MAB3696	53	ATTO550	300	900
5	BCL2	100	Biolegend	658702	69	ATTO647	400	250
6	CD68	KP-1	Biolegend	916104	41	ATTO550	4800	400
6	LAG3	D2G4O	CST	25848SF	42	ATTO647	600	400
7	TCF7/TCF1	Polyclonal	R&D	AF5596	5	ATTO550	600	200
7	CD57	HCD57	MyBioSource	MBS140192	30	ATTO647	150	100
8	CD56	MRQ-42	Cell Marque	custom	29	ATTO550	80	600
8	CD138	B-A38	Novus	NBP3-14568	76	ATTO647	800	300
9	Lambda	MHL-38	Biolegend	316602	26	ATTO550	1500	300
9	NOXA	114C307	Abcam	ab13654	72	ATTO647	800	200
10	CD25	4C9	Cell Marque	custom	24	ATTO550	300	400
10	p53	DO-7	Santa Cruz Biotechnology	sc-47698	52	ATTO647	3200	300
11	CD19	LE-CD19	Bio-RAD	MCA2454	60	ATTO550	150	300
11	FoxP3	236A/E7	Invitrogen	14-4777-82	61	ATTO647	1600	450
12	Podoplanin	D2-40	Biolegend	916606	32	ATTO550	300	800
12	CXCR3 (CD183)	1C6	BD	557183	43	ATTO647	300	300
13	CXCR5	D6L3C	Cell Signaling	custom	62	ATTO550	400	700
13	IRF4	IRF4.3E4	Biolegend	646402	3	ATTO647	1000	200
14	SPARC	AON-5031	Santa Cruz	sc-73472	75	ATTO550	150	700
14	ICOS	D1K2T	CST	39740	74	ATTO647	400	400
15	CD31	C31.3 + C31.7 + C31.10	Novus Bio	NBP2-47785	68	ATTO550	800	600
15	CD163	EDHu-1	Novus	NB110-40686	45	ATTO647	600	200
16	Kappa	L1C1	Cell Marque	short	80	ATTO550	450	300
16	CD4	EPR6855	Abcam	ab181724	20	ATTO647	2400	400
17	CD15	MMA	BD	559045	14	ATTO550	10000	100
17	CD45RO	UCH-L1	NovusBio	NBP2-33104	79	ATTO647	200	300
18	Collagen IV	EPR20966	Abcam	ab226485	38	ATTO550	300	600
18	CD34	QBEnd/10 + HPCA1/763	Novus	NBP2-47909	33	ATTO647	400	300
19	CD20	rIGEL/773	Novus	NBP2-54591	48	ATTO550	1600	150
19	Na+/K+-ATPase	EP1845Y	Abcam	ab283340	67	ATTO647	750	250

20	HLA DR	EPR3692	abcam	ab215985	65	ATTO550	4800	100
20	CD11b	EPR1344	Abcam	ab216445	28	ATTO647	4800	300
21	PD-1	D4W2J	CST	63815SF	23	ATTO550	800	800
21	CD90	EPR3132	Abcam	ab181885	59	ATTO647	3200	200
22	PAX5	D7H5X	CST	93009SF	11	ATTO550	1600	100
22	CD8	C8/144B	NovusBio	NBP2-34588-0.1mg	8	ATTO647	3600	200
23	CD11c	EP1347Y	AbCam	ab216655	49	ATTO550	1600	300
23	CD79a	JBC117	Cell Marque	custom	46	ATTO647	2400	150
24	CD45RA	HI100	Biolegend	304102	63	ATTO550	1600	100
24	Granzyme B	EPR20129-217	Abcam	ab219803	81	ATTO647	3600	75
25	CD3	MRQ-39	Cell Marque	short	77	ATTO550	400	150
25	CD45	2B11 + PD7/26	Novus	NBP2-34528	56	ATTO647	900	150
26	ki-67	B56	BD	556003	2	ATTO550	1600	150
27	CD21	Bu32	Biolegend	354902	21	ATTO550	600	300

Supplementary Table 3: Overview of the antibody panel for the WSIs.

tma_id	wsi_id	entity	entity_class	num_cells	x_pixels	y_pixels
DLBCL_14	DLBCL_1	DLBCL	aggressive	759415	24960	36720
DLBCL_15	DLBCL_2	DLBCL	aggressive	1891857	35520	42480
DLBCL_20	DLBCL_3	DLBCL	aggressive	1417050	35520	56160
DLBCL_6	DLBCL_4	DLBCL	aggressive	2135476	35520	52560
DLBCL_22	DLBCL_5	DLBCL	aggressive	1326811	33600	54000

Supplementary Table 4: Overview of the WSI dataset.
