# Supplementary material for: Spatialproteomics: an interoperable toolbox for analyzing highly multiplexed fluorescence image data
Source: Nat Methods. 2026 Jul 24;23(8):1564–73. doi: 10.1038/s41592-026-03155-1 (PMC13441886; doi:10.1038/s41592-026-03155-1)
Supplement: Supplementary file 2 [file 41592_2026_3155_MOESM2_ESM.pdf]

nature portfolio

Corresponding author(s): Wolfgang Huber, Peter-Martin Bruch, Matthias Meyer-Bender

Last updated by author(s): 02/13/2026

Reporting Summary

Nature Portfolio wishes to improve the reproducibility of the work that we publish. This form provides structure for consistency and transparency in reporting. For further information on Nature Portfolio policies, see our Editorial Policies and the Editorial Policy Checklist.

Statistics

For all statistical analyses, confirm that the following items are present in the figure legend, table legend, main text, or Methods section.

n/a	Confirmed	
☐	☒	The exact sample size (*n*) for each experimental group/condition, given as a discrete number and unit of measurement
☐	☒	A statement on whether measurements were taken from distinct samples or whether the same sample was measured repeatedly
☐	☒	The statistical test(s) used AND whether they are one- or two-sided<br>*Only common tests should be described solely by name; describe more complex techniques in the Methods section.*
☐	☒	A description of all covariates tested
☐	☒	A description of any assumptions or corrections, such as tests of normality and adjustment for multiple comparisons
☐	☒	A full description of the statistical parameters including central tendency (e.g. means) or other basic estimates (e.g. regression coefficient) AND variation (e.g. standard deviation) or associated estimates of uncertainty (e.g. confidence intervals)
☐	☒	For null hypothesis testing, the test statistic (e.g. *F*, *t*, *r*) with confidence intervals, effect sizes, degrees of freedom and *P* value noted<br>*Give P values as exact values whenever suitable.*
☒	☐	For Bayesian analysis, information on the choice of priors and Markov chain Monte Carlo settings
☒	☐	For hierarchical and complex designs, identification of the appropriate level for tests and full reporting of outcomes
☐	☒	Estimates of effect sizes (e.g. Cohen's *d*, Pearson's *r*), indicating how they were calculated

Our web collection on statistics for biologists contains articles on many of the points above.

Software and code

Policy information about availability of computer code

Data collection	Tissue samples were imaged using the Phenocycler Fusion System (Akoya Biosciences) according to the manufacturer's instructions. Acquired raw files were processed into a single qpTIFF file by the Fusion software (version 1.0.7 for TMAs, version 2.2.0 for WSI). A grid defining the locations of the cores within the tissue microarrays was manually constructed in QuPath (v0.4.3) using the built-in TMA de-arrayer.
Data analysis	Data analysis was carried out using the spatialproteomics package (v0.7.4). Raw files were processed by the Fusion software (v 1.0.7 for TMAs, v2.2.0 for WSIs). QuPath v0.4.3 was used for TMA dearraying. External packages that were used: cellpose (v3.0.7 for TMAs, 3.1.0 for WSIs), squidpy 1.6.2, and spatstat v3.1. Spatialdata is available at https://github.com/sagar87/spatialproteomics, and code to reproduce the analysis and figures is available at https://github.com/MeyerBender/spatialproteomics_bnhl.

For manuscripts utilizing custom algorithms or software that are central to the research but not yet described in published literature, software must be made available to editors and reviewers. We strongly encourage code deposition in a community repository (e.g. GitHub). See the Nature Portfolio guidelines for submitting code & software for further information.

Data

Policy information about availability of data

All manuscripts must include a data availability statement. This statement should provide the following information, where applicable:

- Accession codes, unique identifiers, or web links for publicly available datasets
- A description of any restrictions on data availability
- For clinical datasets or third party data, please ensure that the statement adheres to our policy

Raw images and all derived data are available in the BioStudies database (https://www.ebi.ac.uk/biostudies/) under accession number S-BIAD2100 (Sarkans et al., 2018).

Research involving human participants, their data, or biological material

Policy information about studies with human participants or human data. See also policy information about sex, gender (identity/presentation), and sexual orientation and race, ethnicity and racism.

Reporting on sex and gender	We report the sex of the participants, gender was not systematically assessed.
Reporting on race, ethnicity, or other socially relevant groupings	Not systematically assessed due to institutional biobanking policies.
Population characteristics	We report the age at sampling of the patients.
Recruitment	Samples have been obtained from diagnostic biopsies. No systematic bias on sampling is expected.
Ethics oversight	Ethics committee of the medical faculty Heidelberg (ethics vote S-686/2018)

Note that full information on the approval of the study protocol must also be provided in the manuscript.

Field-specific reporting

Please select the one below that is the best fit for your research. If you are not sure, read the appropriate sections before making your selection.

☒ Life sciences ☐ Behavioural & social sciences ☐ Ecological, evolutionary & environmental sciences

For a reference copy of the document with all sections, see nature.com/documents/nr-reporting-summary-flat.pdf

Life sciences study design

All studies must disclose on these points even when the disclosure is negative.

Sample size	Sample size was not predetermined by statistical power calculations. Instead, we queried the samples available in the biobank and assembled the cohort from all available tissues. The sample size is sufficient for the analyses performed within the manuscript, as evidenced by the statistical tests performed in Figure 3a. No associations to clinical outcome is made, as the sample size and heterogeneity of the cohort prevented this.
Data exclusions	Some samples were excluded due to quality issues such as tissue detachment or staining quality.
Replication	Two replicates per patient sample were investigated. Some replicates (total: 28 cores) had to be removed from the analysis due to quality issues (tissue dissociation, marker spillage, or other contamination of the image).
Randomization	No randomization was performed.<br>Tissue samples were collected retrospectively, therefore no active control for clinical covariates were possible. Underlying diagnosis was considered as the main covariate and reported on in Figures 2-4.<br>Covariates during the imaging were minimized since all TMA stainings were performed in one batch with identical staining procedures.
Blinding	During the staining procedures, all samples were stained at the same time. Samples were distributed across all TMAs equally. Sample IDs were not available during the staining procedure.<br>Within the manual thresholding, the person reviewing the samples was blinded as disease group or other sample details were not displayed.<br>In the downstream analysis, blinding was not possible since the cross-entity comparison was the main goal of the analysis.

Reporting for specific materials, systems and methods

We require information from authors about some types of materials, experimental systems and methods used in many studies. Here, indicate whether each material, system or method listed is relevant to your study. If you are not sure if a list item applies to your research, read the appropriate section before selecting a response.

Materials & experimental systems

n/a	Involved in the study
☐	☒ Antibodies
☒	☐ Eukaryotic cell lines
☒	☐ Palaeontology and archaeology
☒	☐ Animals and other organisms
☐	☒ Clinical data
☒	☐ Dual use research of concern
☒	☐ Plants

Methods

n/a	Involved in the study
☒	☐ ChIP-seq
☒	☐ Flow cytometry
☒	☐ MRI-based neuroimaging

Antibodies

Antibodies used: Details on the antibodies used can be found in Supplementary Table 1 and 3.

Validation: Staining specificity was validated in control tissues previous to data acquisition. The manufacturers validation information is provided on the respective websites under the catalogue numbers noted in Supplementary Table 1.

Clinical data

Policy information about clinical studies

All manuscripts should comply with the ICMJE guidelines for publication of clinical research and a completed CONSORT checklist must be included with all submissions.

Clinical trial registration: Data was collected in a standard clinical setting, no clinical trial registration applies.

Study protocol: Data was collected in a standard clinical setting, no clinical trial registration applies.

Data collection: Data was recorded from University of Heidelberg clinical data system throughout 2022.

Outcomes: No outcome measures were used in this analysis.

Plants

Seed stocks: *Report on the source of all seed stocks or other plant material used. If applicable, state the seed stock centre and catalogue number. If plant specimens were collected from the field, describe the collection location, date and sampling procedures.*

Novel plant genotypes: *Describe the methods by which all novel plant genotypes were produced. This includes those generated by transgenic approaches, gene editing, chemical/radiation-based mutagenesis and hybridization. For transgenic lines, describe the transformation method, the number of independent lines analyzed and the generation upon which experiments were performed. For gene-edited lines, describe the editor used, the endogenous sequence targeted for editing, the targeting guide RNA sequence (if applicable) and how the editor was applied.*

Authentication: *Describe any authentication procedures for each seed stock used or novel genotype generated. Describe any experiments used to assess the effect of a mutation and, where applicable, how potential secondary effects (e.g. second site T-DNA insertions, mosiacism, off-target gene editing) were examined.*
