# Supplementary material for: Spatialproteomics: an interoperable toolbox for analyzing highly multiplexed fluorescence image data
Source: Nat Methods. 2026 Jul 24;23(8):1564–73. doi: 10.1038/s41592-026-03155-1 (PMC13441886; doi:10.1038/s41592-026-03155-1)
Supplement: Supplementary file 3 [file 41592_2026_3155_MOESM3_ESM.pdf]

nature portfolio

Peer Review File

Spatialproteomics: an interoperable toolbox for analyzing highly multiplexed fluorescence image data

Corresponding Author: Dr Wolfgang Huber

This file contains all editorial decision letters in order by version, followed by all author rebuttals in order by version.

Attachments originally included by the reviewers as part of their assessment can be found at the end of this file.

Version 0:

Decision Letter:

21st Aug 2025

Dear Dr Huber,

Your Article, "Spatialproteomics: an interoperable toolbox for analyzing highly multiplexed fluorescence image data", has now been seen by 3 reviewers. As you will see from their comments below, although the reviewers find your work of considerable potential interest, they have raised a number of concerns. We continue to be interested in potentially publishing your manuscript in Nature Methods, but would like to evaluate your response to these concerns before we reach a final decision on publication.

We therefore invite you to revise your manuscript to address these concerns particularly those about benchmarking metrics, integration with R packages.

We are committed to providing a fair and constructive peer-review process. Do not hesitate to contact us if there are specific requests from the reviewers that you believe are technically impossible or unlikely to yield a meaningful outcome.

When revising your paper:

* include a point-by-point response to the reviewers and to any editorial suggestions

* please underline/highlight any additions to the text or areas with other significant changes to facilitate review of the revised manuscript

* address the points listed described below to conform to our open science requirements

* ensure it complies with our general format requirements as set out in our guide to authors at www.nature.com/naturemethods

* resubmit all the necessary files by using the link below to access your home page

Link Redacted

Note: This URL links to your confidential account and associated information about manuscripts you may have submitted, or that you are reviewing for us. If you wish to forward this email to co-authors, please delete this link.

We hope to receive your revised paper within three months. If you are substantially delayed, please let us know. In this event, we will still be happy to reconsider your paper at a later date so long as nothing similar has been accepted for publication at Nature Methods or published elsewhere.

OPEN SCIENCE REQUIREMENTS

REPORTING SUMMARY
When revising your manuscript, please update your reporting summary.

Reporting summary: https://www.nature.com/documents/nr-reporting-summary.zip

If your paper includes custom software, we also ask you to complete a supplemental reporting summary.

Software supplement: https://www.nature.com/documents/nr-software-policy.pdf

Please submit these with your revised manuscript. They will be available to reviewers to aid in their evaluation if the paper is re-reviewed. If you have any questions about the checklist, please see http://www.nature.com/authors/policies/availability.html or contact me.

Please note that these forms are dynamic 'smart pdfs' and must therefore be downloaded and completed in Adobe Reader. We will then flatten them for ease of use by the reviewers. If you would like to reference the guidance text as you complete the template, please access these flattened versions at http://www.nature.com/authors/policies/availability.html.

For any revision that includes light microscopy data, we ask our authors to please include a completed light microscopy reporting table [https://www.nature.com/documents/Light_microscopy_reporting_table.xlsx] to ensure the methods are described thoroughly. The table will be available to reviewers and ultimately published should the manuscript be accepted at the journal.

EXTENDED DATA FIGURES
When re-submitting your manuscript, please ensure that any supplementary figures and tables that are crucial to the manuscript's conclusions are converted into Extended Data figures and tables to increase visibility of these data. Extended Data figures and tables are online-only (present in the online PDF and full-text HTML versions of the paper), peer-reviewed display items that provide essential background to the article but are not included in the main article due to space constraints. A maximum of ten Extended Data display items (figures and tables) is permitted.

DATA AVAILABILITY
We strongly encourage you to deposit all new data associated with the paper in a persistent repository where they can be freely and enduringly accessed. We recommend submitting the data to discipline-specific and community-recognized repositories; a list of repositories is provided here: http://www.nature.com/sdata/policies/repositories

All novel DNA and RNA sequencing data, protein sequences, genetic polymorphisms, linked genotype and phenotype data, gene expression data, macromolecular structures, and proteomics data must be deposited in a publicly accessible database, and accession codes and associated hyperlinks must be provided in the "Data Availability" section.

For papers containing bioimaging data, we strongly recommend depositing the data to Bioimage Archive (https://www.ebi.ac.uk/bioimage-archive/). Associated accession codes and hyperlinks should be provided in the "Data Availability" section.

Refer to our data policies here: https://www.nature.com/nature-research/editorial-policies/reporting-standards#availability-of-data

To further increase transparency, we encourage you to provide, in tabular form, the data underlying the graphical representations used in your figures. This is in addition to our data-deposition policy for specific types of experiments and large datasets. For readers, the source data will be made accessible directly from the figure legend. Spreadsheets can be submitted in .xls, .xlsx or .csv formats. Only one (1) file per figure is permitted: thus if there is a multi-paneled figure the source data for each panel should be clearly labeled in the csv/Excel file; alternately the data for a figure can be included in multiple, clearly labeled sheets in an Excel file. File sizes of up to 30 MB are permitted. When submitting source data files with your manuscript please select the Source Data file type and use the Title field in the File Description tab to indicate which figure the source data pertains to.

Please include a "Data availability" subsection in the Online Methods. This section should inform readers about the availability of the data used to support the conclusions of your study, including accession codes to public repositories, references to source data that may be published alongside the paper, unique identifiers such as URLs to data repository entries, or data set DOIs, and any other statement about data availability. At a minimum, you should include the following statement: "The data that support the findings of this study are available from the corresponding author upon request", describing which data is available upon request and mentioning any restrictions on availability. If DOIs are provided, please include these in the Reference list (authors, title, publisher (repository name), identifier, year). For more guidance on how to write this section please see: http://www.nature.com/authors/policies/data/data-availability-statements-data-citations.pdf

CODE AVAILABILITY
Please include a “Code Availability” subsection in the Online Methods which details how your custom code is made available. Only in rare cases (where code is not central to the main conclusions of the paper) is the statement “available upon request” allowed (and reasons should be specified).

We request that you deposit code in a DOI-minting repository such as Zenodo, Gigantum or Code Ocean and cite the DOI in the Reference list. We also request that you use code versioning and provide a license.

For more information on our code sharing policy and requirements, please see:
https://www.nature.com/nature-research/editorial-policies/reporting-standards#availability-of-computer-code

ORCID
Nature Methods is committed to improving transparency in authorship. As part of our efforts in this direction, we are now requesting that all authors identified as ‘corresponding author’ on published papers create and link their Open Researcher and Contributor Identifier (ORCID) with their account on the Manuscript Tracking System (MTS), prior to acceptance. This applies to primary research papers only. ORCID helps the scientific community achieve unambiguous attribution of all scholarly contributions. You can create and link your ORCID from the home page of the MTS by clicking on ‘Modify my Springer Nature account’. For more information please visit please visit www.springernature.com/orcid.

Please do not hesitate to contact me if you have any questions or would like to discuss these revisions further. We look forward to seeing your revised manuscript and thank you for the opportunity to consider your work.

Best regards,
Madhura

Madhura Mukhopadhyay, PhD
Senior Editor
Nature Methods

Reviewers' Comments:

Reviewer #1 (Remarks to the Author):

This manuscript introduces spatialproteomics, a Python toolbox for end-to-end analysis of multiplexed fluorescence imaging. The tool is original and well-integrated with the scverse ecosystem, offering consistent data structures, efficient large-image handling, and modular analysis steps. The authors validate it on a substantial BNHL dataset and provide clear methodological details. Results are convincing, though benchmarking with other tools and more discussion on QC and thresholding would strengthen the work. Overall, the study is methodologically sound, relevant, and a valuable addition to spatial omics.
I have some comments, please see the attachment.

Reviewer #1 (Remarks on code availability):

Overall, the code is userfriendly and reproducible, but some reference data are not provided. Moreover, the documentation needs more details for a new user.

Reviewer #2 (Remarks to the Author):

A. Summary of the key results
In the manuscript by Meyer-Bender et al., the authors present a pipeline for multiplexed imaging data, spatial proteomics. The proposed pipeline is based on xarray and dask, which allows users to process large-scale images and perform cell segmentation, image processing, marker quantification, cell type classification, and neighborhood analysis with synchronization of shared coordinates across data. Storing processed data as zarr files enables users to load data in a memory-efficient way. In the manuscript by Meyer-Bender et al., the authors present a pipeline for multiplexed imaging data, spatialproteomics. The proposed pipeline is based on xarray and dask, which allows users to process large-scale images and perform cell segmentation, image processing, marker quantification, cell type classification, and neighborhood analysis with synchronization of shared coordinates across data. Storing data as zarr files enables users to load and process data in a memory-efficient way. Furthermore, the authors implemented export functions to AnnData and spatialdata to improve interoperability between spatial analysis tools. The authors demonstrate the capabilities of spatialproteomics by analyzing

gigapixel-scale tissue microarray and whole-slide images with 56 markers, and performing image preprocessing, cell segmentation, cell annotation, and spatial analysis on millions of cells.

B. Originality and significance
The authors have developed a unified pipeline called spatialproteomics that enables end-to-end analysis from raw image segmentation to cell type annotation and neighborhood analysis. Furthermore, by integrating these results into a single object, the authors have streamlined data handling that previously required separate generation and storage for each analysis step using conventional libraries.

C. Data & methodology
The principles underlying the pipeline construction are well-articulated, and the steps involved in the image analysis workflow are delineated. This clarity largely stems from the fact that these procedures follow well-established and widely recognized methods and libraries for multiplexed image analysis.

D. Appropriate use of statistics and treatment of uncertainties
The statistical analysis appear to be conducted appropriately.

E. Conclusions: robustness, validity, reliability
The conclusion would be strengthened by more clearly highlighting the key advantages of this spatialproteomics pipeline in comparison to existing tools, such as MCMICRO.

F. Suggested improvements: experiments, data for possible revision
1. In multiplex analysis, it is essential to be able to interactively compare images and data during interpretation. However, based on the manuscript and the documentation provided by the authors (https://sagar87.github.io/spatialproteomics/index.html), the extent of interactivity offered by the proposed spatialproteomics framework remains unclear. Ideally, the pipeline should enable interactive plotting—from raw image visualization to spatial analysis results—using tools such as Napari. If such functionality is already implemented, it would be beneficial to clearly describe and emphasize it in the main text.

2. The authors conduct an interesting spatial analysis using the spatstat package in their manuscript. However, since spatstat is an R package, it is likely difficult to directly analyze data generated by the spatialproteomics framework without additional processing. To enhance one of the key strengths of spatialproteomics —its seamless integration with other analytical tools—it would be highly desirable to enable spatstat-based analyses to be performed directly within the spatialproteomics environment.

3. In Fig. 4a, the authors adjusted differences in marker intensities across tissues by subtracting a second image that was a Gaussian-blurred version of the original. Is this processing step implemented as a function within the spatialproteomics framework? Additionally, is the corresponding code available somewhere?

4. As the authors themselves mention in the Discussion, several analysis pipelines with similar concepts to the proposed spatialproteomics already exist. It would be helpful to include a comparative table summarizing the differences and highlighting the advantages of spatialproteomics.

5. Supplementary Figures 4 and 5 were not cited in the main text.

6. It would be desirable for the authors to discuss the future development directions of SpatialProteomics in the Discussion section. For example, it would be valuable to address how the framework plans to handle the increasingly important integration of multiplexed fluorescence imaging data with spatial transcriptomics data.

G. References
The references appear appropriate, with all major prior work adequately cited.

H. Clarity and context
The manuscript is well-written and clearly articulated.

Reviewer #3 (Remarks to the Author):

I co-reviewed this manuscript with one of the reviewers who provided the listed reports. This is part of the Nature Methods initiative to facilitate training in peer review and to provide appropriate recognition for Early Career Researchers who co-review manuscripts.<</sub>/sup>

Reviewer #3 (Remarks on code availability):

I encountered an error when attempting to install spatialproteomics alongside other tools such as Cellpose using
pip install spatialproteomics[cellpose]
on an Apple Silicon Mac. In contrast, the installation proceeded without issues on a Linux (Ubuntu) system.

I attempted to test the functionality of spatialproteomics by following the tutorial; however, it was unclear how to obtain the image data and other

resources used in the tutorial.To ensure the reproducibility of the tutorial, the authors should clearly specify the source or method for downloading the image data used in the tutorial.

Version 1:

Decision Letter:

Our ref: NMETH-A61616A

8th Dec 2025

Dear Wolfgang,

Thank you for submitting your revised manuscript "Spatialproteomics: an interoperable toolbox for analyzing highly multiplexed fluorescence image data" (NMETH-A61616A). It has now been seen by the original referees and their comments are below. The reviewers find that the paper has improved in revision, and therefore we'll be happy in principle to publish it in Nature Methods, pending minor revisions to satisfy the referees' final requests and to comply with our editorial and formatting guidelines.

We are now performing detailed checks on your paper and will send you a checklist detailing our editorial and formatting requirements within two weeks or so. Please do not upload the final materials and make any revisions until you receive this additional information from us.

TRANSPARENT PEER REVIEW
Nature Methods offers a transparent peer review option for new original research manuscripts submitted from 17th February 2021. We encourage increased transparency in peer review by publishing the reviewer comments, author rebuttal letters and editorial decision letters if the authors agree. Such peer review material is made available as a supplementary peer review file. **Please state in the cover letter 'I wish to participate in transparent peer review' if you want to opt in, or 'I do not wish to participate in transparent peer review' if you don't.** Failure to state your preference will result in delays in accepting your manuscript for publication.

Please note: we allow redactions to authors' rebuttal and reviewer comments in the interest of confidentiality. If you are concerned about the release of confidential data, please let us know specifically what information you would like to have removed. Please note that we cannot incorporate redactions for any other reasons. Reviewer names will be published in the peer review files if the reviewer signed the comments to authors, or if reviewers explicitly agree to release their name. For more information, please refer to our FAQ page.

ORCID
IMPORTANT: Non-corresponding authors do not have to link their ORCIDs but are encouraged to do so. Please note that it will not be possible to add/modify ORCIDs at proof. Thus, please let your co-authors know that if they wish to have their ORCID added to the paper they must follow the procedure described in the following link prior to acceptance: https://www.springernature.com/gp/researchers/orcid/orcid-for-nature-research

Author names using non-Roman characters
Nature Portfolio journals can support presentation of author names using non-Roman characters in the HTML version of the article. If you wish to, please include author names in parentheses after the Roman-character spelling; see example online here. Currently supported scripts are: Arabic, Chinese, Cyrillic, Devanagari, Greek, Hebrew, Hangul, Japanese and Persian. You will be asked to verify the rendering is correct at proof stage.

Thank you again for your interest in Nature Methods. Please do not hesitate to contact me if you have any questions. We will be in touch again soon.

Sincerely,
Madhura

Madhura Mukhopadhyay, PhD
Senior Editor
Nature Methods

Reviewer #1 (Remarks to the Author):

I still have some follow-up questions:

On Question 3,I do appreciate the authors' effort to add a quantitative evaluation of inter-annotator robustness and to compare manual gating with argmax-based labels and astir (Sup. Fig. 4). However, the current response still leaves some ambiguity regarding the impact of threshold choice on the final results. At present, the argument is mainly based on high Cohen's kappa between the three human annotators, but it remains unclear like: (i) how different the actual marker thresholds chosen by each annotator were, and (ii) to what extent reasonable perturbations of these thresholds would affect cell-type proportions or downstream spatial statistics.
In other words, high inter-annotator agreement may partly reflect shared prior expectations and a common gating template, rather than demonstrating that threshold choice has only a minor effect on the outcome.

I would therefore encourage the authors to briefly clarify these points, by reporting the range of thresholds chosen by different annotators for key markers, and adding a simple sensitivity analysis showing how varying selected thresholds within a plausible range affects key quantitative readouts. In addition, the response does not fully address the original question about more data-driven thresholding strategies or diagnostic tools. It would be helpful if the authors could explicitly state whether they plan to implement more systematic threshold selection methods or at least provide recommended diagnostic plots in the documentation. Even a concise statement about current limitations and future plans would make the workflow's assumptions more transparent to users.

On Question 4, the new notebook illustrating the integration of IMC, MICS and CODEX data at the cell-type level is a useful addition. Nevertheless, the response still does not fully address the original concern about cross-platform marker harmonization and multi-batch alignment. As far as I understand, spatialproteomics assumes that each dataset has already been independently normalized and cell-typed, and then operates on a labelled point process, like cell coordinates + cell-type labels for integration.
If this interpretation is correct, I would suggest that the authors make this assumption explicit both in the manuscript and in the documentation: spatialproteomics currently does not provide dedicated functionality for harmonizing partially overlapping marker panels or aligning marker intensities

across platforms, and instead relies on users to define comparable cell-type labels upstream. Clarifying this limitation and, if possible, briefly indicating whether marker-level harmonization is a planned future extension would help manage user expectations and make the scope of the toolkit more precise.

Reviewer #2 (Remarks to the Author):

The authors have responded adequately to my concerns.

Reviewer #3 (Remarks to the Author):

I co-reviewed this manuscript with one of the reviewers who provided the listed reports. This is part of the Nature Methods initiative to facilitate training in peer review and to provide appropriate recognition for Early Career Researchers who co-review manuscripts.

Version 2:

Decision Letter:

5th Jun 2026

Dear Wolfgang,

I am pleased to inform you that your Article, "Spatialproteomics: an interoperable toolbox for analyzing highly multiplexed fluorescence image data", has now been accepted for publication in Nature Methods. The received and accepted dates will be Jun 24, 2025 and Jun 05, 2026. This note is intended to let you know what to expect from us over the next month or so, and to let you know where to address any further questions.

Over the next few weeks, your paper will be copyedited to ensure that it conforms to Nature Methods style. Once your paper is typeset, you will receive an email with a link to choose the appropriate publishing options for your paper and our Author Services team will be in touch regarding any additional information that may be required. It is extremely important that you let us know now whether you will be difficult to contact over the next month. If this is the case, we ask that you send us the contact information (email, phone and fax) of someone who will be able to check the proofs and deal with any last-minute problems.

After the grant of rights is completed, you will receive a link to your electronic proof via email with a request to make any corrections within 48 hours. If, when you receive your proof, you cannot meet this deadline, please inform us at rjsproduction@springernature.com immediately.

Authors may need to take specific actions to achieve compliance with funder and institutional open access mandates. If your research is supported by a funder that requires immediate open access (e.g. according to Plan S principles or the NIH public access policy) then you should select the gold OA route, and we will direct you to the compliant route where possible. Because authors warrant under our subscription licensing terms that they haven't committed to licensing any version of their article under a licence inconsistent with the terms of our agreement – including the applicable embargo period – publication under the subscription model isn't suitable for authors whose funders require no embargo.

If you have any questions about our publishing options, costs, Open Access requirements, or our legal forms, please contact ASJournals@springernature.com

If you have posted a preprint on any preprint server, please ensure that the preprint details are updated with a publication reference, including the DOI and a URL to the published version of the article on the journal website.

You may wish to make your media relations office aware of your accepted publication, in case they consider it appropriate to organize some internal or external publicity. Once your paper has been scheduled you will receive an email confirming the publication details. This is normally 3-4 working days in advance of publication. If you need additional notice of the date and time of publication, please let the production team know when you receive the proof of your article to ensure there is sufficient time to coordinate. Further information on our embargo policies can be found here: https://www.nature.com/authors/policies/embargo.html

To assist our authors in disseminating their research to the broader community, our SharedIt initiative provides you with a unique shareable link that will allow anyone (with or without a subscription) to read the published article. Recipients of the link with a subscription will also be able to download and print the PDF.

As soon as your article is published, you will receive an automated email with your shareable link.

If you are active on Twitter/X or Bluesky, please e-mail me your and your coauthors' handles so that we may tag you when the paper is published.

You can now use a single sign-on for all your accounts, view the status of all your manuscript submissions and reviews, access usage statistics for your published articles and download a record of your refereeing activity for the Nature journals.

Please note that you and any of your coauthors will be able to order reprints and single copies of the issue containing your article through Nature Portfolio's reprint website, which is located at http://www.nature.com/reprints/author-reprints.html. If there are any questions about reprints please send an email to author-reprints@nature.com and someone will assist you.

Please feel free to contact me if you have questions about any of these points.

Best regards,
Madhura

Madhura Mukhopadhyay, PhD
Senior Editor
Nature Methods

** Visit the Springer Nature Editorial and Publishing website at www.springernature.com/editorial-and-publishing-jobs for more information about our career opportunities. If you have any questions please click here.**

Open Access This Peer Review File is licensed under a Creative Commons Attribution 4.0 International License, which permits use, sharing, adaptation, distribution and reproduction in any medium or format, as long as you give appropriate credit to the original author(s) and the source, provide a link to the Creative Commons license, and indicate if changes were made.
In cases where reviewers are anonymous, credit should be given to 'Anonymous Referee' and the source.
The images or other third party material in this Peer Review File are included in the article's Creative Commons license, unless indicated otherwise in a credit line to the material. If material is not included in the article's Creative Commons license and your intended use is not permitted by statutory regulation or exceeds the permitted use, you will need to obtain permission directly from the copyright holder.
To view a copy of this license, visit https://creativecommons.org/licenses/by/4.0/

Response to the reviewers

We thank the editor and the reviewers for the time that they invested into our work, for their constructive suggestions on its better presentation and overall for their positive feedback and encouragement on our package *spatialproteomics*. Based on this input, we have (i) further improved the documentation, especially in regard to the interoperability with other analysis tools, both in *Python* and *R*, (ii) benchmarked the memory usage and runtime with respect to dataset size, and (iii) adapted the manuscript to include the suggestions made by the reviewers. Moreover, following a suggestion by Reviewer 1, we added a quantitative study on the robustness of cell type annotation across three users and the comparison to automated cell type prediction, on what we consider a typical dataset and use case.

Overall, we believe the changes have further improved the presentation of our toolbox and should help readers better understand its value for their own analyses of highly multiplexed imaging data. We provide detailed responses to the reviewers' comments below.

Reviewer 1

We thank the reviewer for their constructive comments, which have substantially improved both the documentation of the software, and the presentation of *spatialproteomics* in the manuscript. Based on the suggestions, we have improved the documentation on interoperability, including that with R packages, downstream analysis, customizability, and also show how users can apply *spatialproteomics* to different modalities (such as IMC and MICS) with non-identical marker panels. We have also benchmarked the performance gains and memory savings obtained by the usage of *Dask* and *zarr*, compared to reading the data from *qptiff* files and performing analyses in a sequential instead of a parallelized manner. Finally, we assessed the inter-annotator robustness of our threshold-based cell type prediction.

1. The documentation of spatialproteomics is clear and well-structured, with tested code examples and easy-to-follow tutorials. The guides on image processing and cell type annotation are especially user-friendly. However, key functions like neighborhood analysis and integration with downstream tools (e.g., squidpy in Python or spatstat in R) are either shown only in separate examples or not covered in detail. Including these features in a unified workflow would improve usability, especially for new users unfamiliar with spatial analysis or the broader scverse ecosystem.

We thank the reviewer for this positive assessment. Following their suggestion, we have added a notebook which shows how users can use spatialproteomics objects as a starting point to carry out downstream analysis with *squidpy* and *spatstat*. This notebook can be found here[1].

2. The authors provide a dedicated section on interoperability, with support for exporting to Zarr, CSV, AnnData, and SpatialData, making it easy to integrate with Python tools like scanpy and squidpy. However, there is no mention of support for R-based frameworks such as Seurat, which are still widely used in spatial transcriptomics. Although spatstat in R is

[1] https://sagar87.github.io/spatialproteomics/notebooks/DownstreamAnalysis.html

used for spatial statistics like Ripley's K, it's unclear whether this is part of an integrated workflow or run separately. Including an example that bridges into R, or clarifying plans for R compatibility, would make the toolkit more versatile.

Spatialproteomics provides a variety of export options to language-agnostic data structures such as *spatialdata* or csv files (for appropriate subsets of the data). An example of how this enables R compatibility is shown in the "Downstream Analysis" notebook[2]. In addition, we have added a section on how to load the spatialproteomics data into *Seurat* here[3].

3. The current marker thresholding strategy for cell type classification relies on manual tuning, starting from fixed quantiles and adjusted by visual inspection. While this offers flexibility, it introduces subjectivity and may limit reproducibility. It would be helpful if the authors could clarify whether they plan to include more data-driven thresholding methods (e.g., adaptive thresholding or mixture models), or at least provide heuristics or diagnostic plots to guide users. This would reduce the need for trial-and-error, especially for users with limited prior knowledge. Additionally, since the annotation is based on decision trees with quantile thresholds, some form of benchmarking—such as consistency metrics, F1 scores, or agreement with expert labels—would help justify threshold choices and improve transparency.

We thank the reviewer for highlighting this important point. Cell type prediction is a complex bioinformatic challenge that is highly contingent on the dataset and biological questions posed. While here we present a manual thresholding approach, a broad range of cell type annotation tools are supported in *spatialproteomics*. To highlight this flexibility, we have performed cell type annotation using *astir* and compared it to the manual thresholding previously reported, as well as two additional versions of the same manual thresholding approach, performed by independent human annotators.

This analysis highlights the robustness and comparability between different researchers. Details are shown in **Sup. Fig. 4**. The ideal tool and methodology for cell type annotation however has to be picked individually for each dataset, which is why the flexibility[4] offered by *spatialproteomics* is essential for this application. We added the following text to the manuscript:

"Since our cell type assignment was preceded by a manual selection of thresholds, we aimed to control inter-annotator variability. To this end, we selected 40 cores (20 FL, 20 DLBCL) and asked three different human annotators to perform the threshold selection independently (**Sup. Table 5, Sup. Table 7**). We also considered two alternative classification methods: assigning cell types based upon which marker had the highest mean expression in a cell without any thresholding and *astir*, a fully automatic method (Geuenich *et al.*, 2021). The classifications from the three annotators agreed more with one another than with the assignment from the raw intensities or *astir* (**Sup. Fig. 4a,b**). The average thresholded marker profiles per cell type looked very similar across annotators (**Sup. Fig. 4c**). A major source of discrepancy between the human annotations and *astir* was the fact that *astir* was given the option of assigning the cell type "Other" for 16.7% of all cells,

[2] https://sagar87.github.io/spatialproteomics/notebooks/DownstreamAnalysis.html
[3] https://sagar87.github.io/spatialproteomics/notebooks/Interoperability.html#Exporting-to-Seurat
[4] https://sagar87.github.io/spatialproteomics/notebooks/Customizability.html#Custom-Cell-Type-Prediction

whereas the human-thresholded gating tree always assigns one of the predefined cell types (**Sup. Fig. 4d**)."

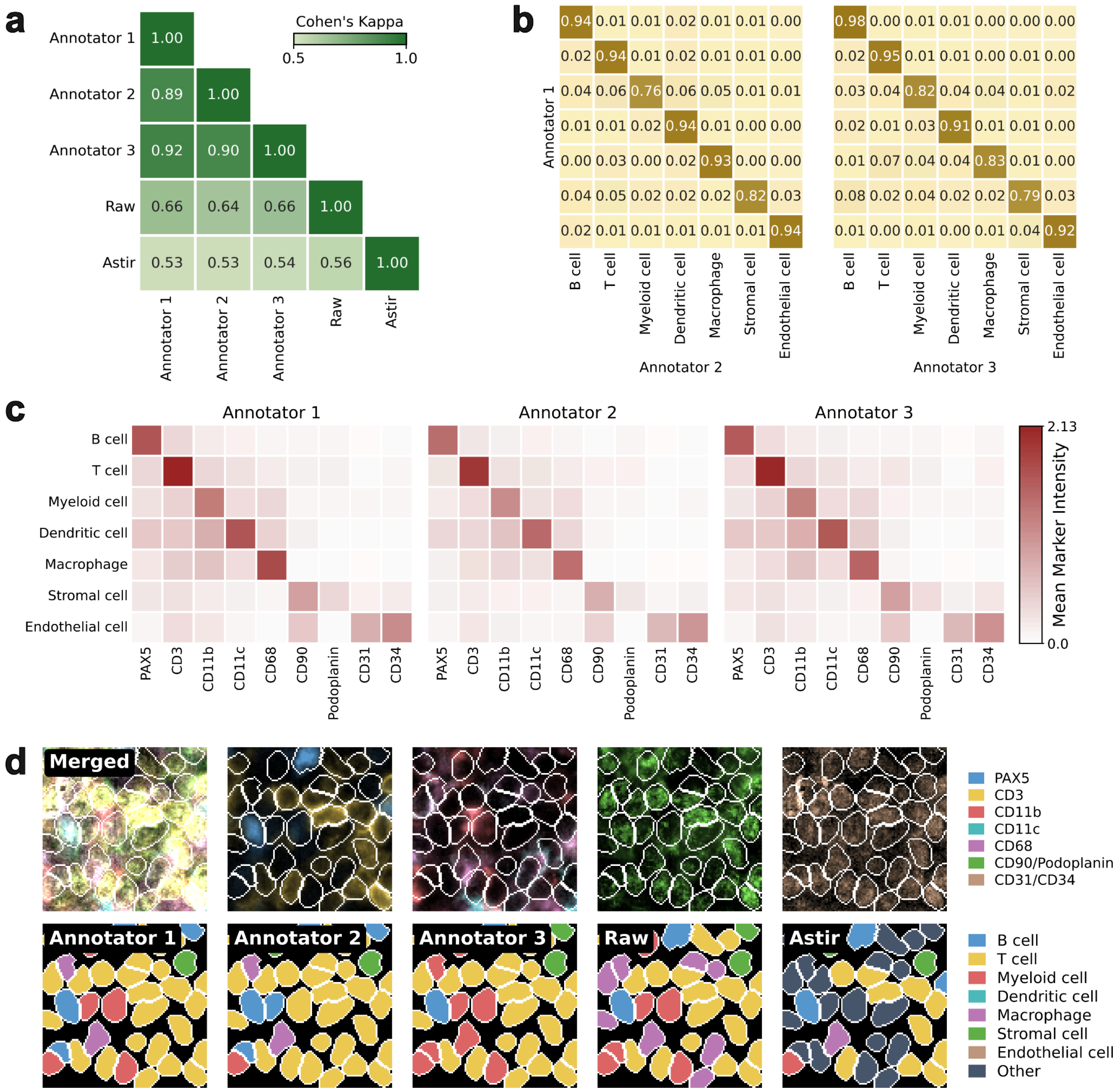

Supplementary Figure 4: Manual thresholding was robust across independent annotators and entities. **a**, Cohen's Kappa between three independent human annotators, predictions on the unthresholded images, and *astir*. Human annotators agreed more with one another than with the fully automated approaches. **b**, Confusion matrices show high agreement between annotators. **c**, Mean marker intensities for each cell type after thresholding. Despite the thresholding being performed independently, the resulting marker profiles looked similar across annotators. **d**, Exemplary cell type prediction and the corresponding markers. The top row also shows the disentangled marker intensities for better visibility.

4. The current version of spatialproteomics seems best suited for single-batch datasets with consistent marker panels, as demonstrated in the BNHL use case. However, it is unclear how the package handles cross-sample integration when marker sets differ, such as in multi-batch designs or across platform versions. Even if not currently supported, it would be

useful for the authors to clarify whether marker harmonization or multi-sample alignment is a planned feature. Furthermore, demonstrating the toolkit's ability to integrate or compare datasets from different imaging modalities (e.g., CODEX, IMC, MIBI), which often share only partial marker overlap and differ in data characteristics, would strengthen its value. Clear guidance or examples on how such cross-platform analyses could be approached using spatialproteomics would significantly broaden its applicability.

Integration of data produced with non-identical marker sets or from different imaging modalities is achieved in *spatialproteomics* once the data are converted into a labelled point process, i.e., centroid coordinates and cell type label for each cell. We note that analysts will want to consider whether or to what extent the different modalities resolve the same, or close-enough, cell type categorizations, and how the detection/classification sensitivities compare between them. We have therefore added a notebook[5] to the documentation that shows an exemplary integration of data from IMC, MICS, and CODEX.

5. While the authors highlight the use of “xarray”and “dask” to support scalable data handling, the manuscript lacks a systematic evaluation of performance in terms of runtime, memory usage, and hardware requirements. It would be valuable to include concrete metrics—such as peak memory, processing time, and CPU/GPU specs—especially for full whole-slide images or large TMA cohorts. This information would help guide users with limited computing resources. Additionally, beyond the session details in the documentation, a more thorough benchmarking analysis showing how performance scales with image size, number of channels, or cell count would strengthen the claims of scalability and robustness, and make the toolkit more practical for broader use cases.

We have benchmarked runtime and peak memory requirements, the results of which are assembled in **Sup. Fig. 2**. In short, the lazy-loading functionality of *spatialproteomics* enables the analysis of large datasets with moderate resource requirements, as both the channels and image area are subsetted. While this has a cost in case the entire dataset is loaded, a subset of markers can be loaded while still using less memory than by directly loading the entire dataset. Thereby, most usage scenarios profit off this data handling strategy. Alternatively, lazy-loading can be skipped by using *xr.load_dataset()* in usage scenarios where the entire image is needed. This is detailed in the documentation[6].

We also added a small paragraph to the main text, explaining the advantages of using *xarray* in more detail. The usage of GPUs mainly speeds up segmentation algorithms, which we did not explicitly benchmark, since their use is very dependent on the research question as well as the hardware at hand. We have also added a small section on parallelization with *Dask* and memory optimization to the FAQ[7].

“On disk, objects are stored as *zarr* files, so that users can load image slices, segmentation masks, quantifications, cell type assignments and other possibly interesting data without needing to bring the whole object into working memory. This design enables analysis of large datasets on compute infrastructure that would otherwise fail due to memory constraints (**Sup. Fig. 2a,b**). Since *spatialproteomics* is based on *xarray*, users can also make full use of

[5] https://sagar87.github.io/spatialproteomics/notebooks/MultiTechnologyIntegration.html
[6] https://sagar87.github.io/spatialproteomics/notebooks/FAQ.html#How-can-I-optimize-my-memory-usage?
[7] https://sagar87.github.io/spatialproteomics/notebooks/FAQ.html

Dask's parallelization capabilities, which can reduce runtime compared to sequential processing of many samples (**Sup. Fig. 2c,d**). "

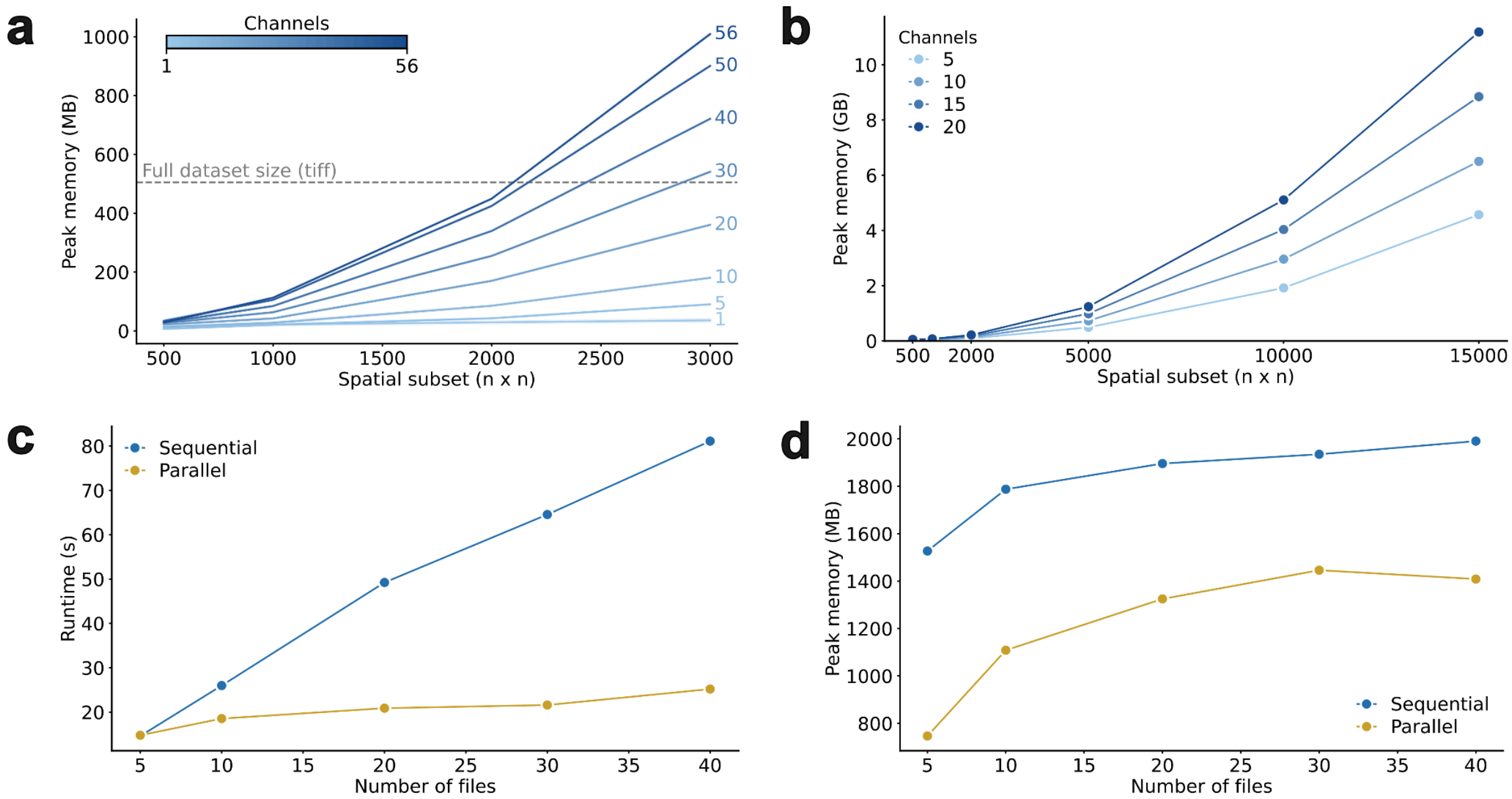

Supplementary Figure 2: Lazy loading with *Dask* enables memory efficient subsetting of cores. **a,** Loading of different numbers of markers and spatial subsets across 40 TMA cores. When subsetting to a smaller area, or selecting a limited amount of markers, lazy loading conserves runtime compared to reading the full dataset into memory. It is however notable that when loading the full data into memory (and thereby converting the *Dask*-backed object into a *numpy* array), the peak memory briefly doubles. **b,** Loading of a WSI with over 100GB on disk (35,520 by 56,160 pixels, 53 channels). Subsetting by markers or a region reduces memory requirements and enables analysis on low-memory machines. **c,** Parallelization with *Dask* across 16 workers enables faster runtimes when analyzing many samples at once (here: quantification and arcsinh-transform). **d,** Parallelization across 16 workers does not come at the cost of increased memory. Instead, the peak memory per worker is reduced.

6. The package usefully integrates several leading tools for segmentation and spatial analysis (e.g., cellpose, stardist, mesmer, squidpy) through a consistent and user-friendly interface, which is a major strength. However, given the rapid development of new methods in this field, it would be helpful if the authors could clarify how easily users can incorporate alternative tools. For instance, if a new segmentation model is developed, can users load custom segmentation masks or label images into the spatialproteomics object for downstream analysis? Likewise, can existing neighborhood graphs or clustering steps be replaced with user-defined logic, such as GNN-based representations? Even if full modularity is not intended, guidance on how to connect external outputs to the pipeline—via format compatibility or accessor injection—would support broader adoption and adaptability in evolving workflows.

We thank the reviewer for highlighting this need for flexibility in a constantly developing analysis landscape. With this in mind, we have designed *spatialproteomics* to be fully

customizable. A specific notebook on customizability[8] has been added to the documentation. It outlines how users can load custom segmentations, cell type annotations, GNN embeddings, and apply custom image processing methods. Thereby, *spatialproteomics* can be adapted to virtually any new analysis method in these steps. We have also added a small section about customizability to the text:

“Customizability is a key feature of *spatialproteomics*. It provides wrappers for established tools and is ready to interoperate with new methods as the field evolves. Its well-documented and accessible data representation enables users to programmatically extend all aspects of functionality, e.g., for segmentation, cell phenotyping, network analysis or spatial statistics.”

Reviewer 2

We thank the reviewer for the constructive comments, which have substantially improved both the documentation as well as the presentation of *spatialproteomics* in the manuscript. Based on the suggestions, we have improved the documentation with respect to interactive data analysis with *napari*, as well as interoperability with R. We have furthermore added a table to the manuscript comparing *spatialproteomics* to existing tools for the analysis of highly multiplexed fluorescence imaging data. Lastly, we have added a paragraph on the future development of *spatialproteomics*.

A. Summary of the key results
In the manuscript by Meyer-Bender et al., the authors present a pipeline for multiplexed imaging data, spatial proteomics. The proposed pipeline is based on xarray and dask, which allows users to process large-scale images and perform cell segmentation, image processing, marker quantification, cell type classification, and neighborhood analysis with synchronization of shared coordinates across data. Storing processed data as zarr files enables users to load data in a memory-efficient way. In the manuscript by Meyer-Bender et al., the authors present a pipeline for multiplexed imaging data, spatialproteomics. The proposed pipeline is based on xarray and dask, which allows users to process large-scale images and perform cell segmentation, image processing, marker quantification, cell type classification, and neighborhood analysis with synchronization of shared coordinates across data. Storing data as zarr files enables users to load and process data in a memory-efficient way. Furthermore, the authors implemented export functions to AnnData and spatialdata to improve interoperability between spatial analysis tools. The authors demonstrate the capabilities of spatialproteomics by analyzing gigapixel-scale tissue microarray and whole-slide images with 56 markers, and performing image preprocessing, cell segmentation, cell annotation, and spatial analysis on millions of cells.

B. Originality and significance
The authors have developed a unified pipeline called spatialproteomics that enables end-to-end analysis from raw image segmentation to cell type annotation and neighborhood analysis. Furthermore, by integrating these results into a single object, the authors have streamlined data handling that previously required separate generation and storage for each analysis step using conventional libraries.

[8] https://sagar87.github.io/spatialproteomics/notebooks/Customizability.html

C. Data & methodology
The principles underlying the pipeline construction are well-articulated, and the steps involved in the image analysis workflow are delineated. This clarity largely stems from the fact that these procedures follow well-established and widely recognized methods and libraries for multiplexed image analysis.

D. Appropriate use of statistics and treatment of uncertainties
The statistical analysis appears to be conducted appropriately.

E. Conclusions: robustness, validity, reliability
The conclusion would be strengthened by more clearly highlighting the key advantages of this spatialproteomics pipeline in comparison to existing tools, such as MCMICRO.

We thank the reviewer for their positive assessment of our work. To contextualize our work in comparison to existing tools, we assembled **Supplementary Table 7** comparing the different methods available for multiplexed image analysis.

**Package**	spatialproteomics	sopa	SPACEc	MCMICRO	SIMPLI	imcRtools/steinbock
**Language**	Python	Python, Snakemake	Python	Nextflow	Nextflow	R, Python
**Data Format**	Xarray or spatialdata	spatialdata	Tif, csv, ...	Tif, csv, ...	Tif, csv, ...	SpatialExperiment
**Enforced Synchronization of Shared Coordinates**	Yes	No	No	No	No	Yes
**Segmentation**	Cellpose, Mesmer, Stardist	Cellpose	Cellpose, Mesmer	UnMICST, S3segmenter	CellProfiler, Stardist	Cellpose, Mesmer, CellProfiler
**Image Processing**	Fully customizable	None	None	None	Thresholding	Hot Pixel Removal
**Protein Quantification**	Fully customizable	Mean, min, max	Mean	MCQuant	CellProfiler	Mean
**Expression Matrix Processing**	Z-score, Double Z-score, MinMax, ArcSin, Clip	Z-score, Spillover Correction	Z-score, Double Z-score, MinMax, ArcSin	Log1p, Rescale, ComBat	CellProfiler	Spillover Compensation
**Cell Phenotyping**	Astir, Gating (Argmax, Binarization)	Argmax	SVM, STELLAR, Clustering	SCIMAP (Clustering, Gating)	Clustering, Thresholding	Random Forest, Clustering
**Neighborhood Analysis**	Clustering	NA	Clustering	SCIMAP (Spatial LDA, Spatial Lag)	Clustering	Clustering, lisaClust
**Interactivity**	Napari	Napari	TissUUmaps	Napari (CyLinter), Minerva	NA	Napari (napari-imc), cytoviewer/cytomapper

Supplementary Table 8: Comparison of different tools for analyzing highly multiplexed immunofluorescence images.

F. Suggested improvements: experiments, data for possible revision

1. In multiplex analysis, it is essential to be able to interactively compare images and data during interpretation. However, based on the manuscript and the documentation provided by the authors (https://sagar87.github.io/spatialproteomics/index.html), the extent of interactivity offered by the proposed spatialproteomics framework remains unclear. Ideally, the pipeline should enable interactive plotting—from raw image visualization to spatial analysis results—using tools such as Napari. If such functionality is already implemented, it would be beneficial to clearly describe and emphasize it in the main text.

As highlighted by the reviewer, an interactive and iterative process of visualization and data processing is key to analyse these data types. *Spatialproteomics* includes a plotting module built on *matplotlib*, enabling users to customize visualizations programmatically. For those who prefer a graphical interface, integration with *napari* is supported. To facilitate this, we have extended the documentation as well as the manuscript to include a more thorough description of how the interaction between *spatialproteomics* and *napari-spatialdata* works. The corresponding notebook can be found here[9].

2. The authors conduct an interesting spatial analysis using the spatstat package in their manuscript. However, since spatstat is an R package, it is likely difficult to directly analyze data generated by the spatialproteomics framework without additional processing. To enhance one of the key strengths of spatialproteomics —its seamless integration with other analytical tools—it would be highly desirable to enable spatstat-based analyses to be performed directly within the spatialproteomics environment.

The integration of different analysis tools and environments is a key component of *spatialproteomics*. To further highlight how the interface with R can be leveraged, we have added a notebook to the documentation, which specifically shows how users can use packages such as *spatstat* to perform downstream analysis. This notebook can be found here[10].

3. In Fig. 4a, the authors adjusted differences in marker intensities across tissues by subtracting a second image that was a Gaussian-blurred version of the original. Is this processing step implemented as a function within the spatialproteomics framework? Additionally, is the corresponding code available somewhere?

In addition to the code used for this particular analysis, which can be found here[11], we have added a section on this in the documentation[12]. We wrote a custom function to perform this processing, using the cv2 package, and then used the `pp.apply()` method from spatialproteomics to apply this to the images.

4. As the authors themselves mention in the Discussion, several analysis pipelines with similar concepts to the proposed spatialproteomics already exist. It would be helpful to

[9] https://sagar87.github.io/spatialproteomics/notebooks/Interactivity.html
[10] https://sagar87.github.io/spatialproteomics/notebooks/DownstreamAnalysis.html
[11] https://github.com/MeyerBender/spatialproteomics_bnhl/blob/main/wsi_analysis/scripts/07_threshold_and_predict_cts.py
[12] https://sagar87.github.io/spatialproteomics/notebooks/Customizability.html#Custom-Image-Processing

include a comparative table summarizing the differences and highlighting the advantages of spatialproteomics.

As described above, we have assembled a method comparison in **Supplementary Table 8**.

5. Supplementary Figures 4 and 5 were not cited in the main text.

We have added the missing references to the main text. We also noticed that the PMBCL samples were missing from these figures, so we added them in the revised version of the manuscript.

6. It would be desirable for the authors to discuss the future development directions of SpatialProteomics in the Discussion section. For example, it would be valuable to address how the framework plans to handle the increasingly important integration of multiplexed fluorescence imaging data with spatial transcriptomics data.

We have added a paragraph outlining future directions to the ”Discussion” section:

“As spatial omics technologies continue to develop, *spatialproteomics* is designed to evolve alongside. While the package already allows users to incorporate external tools into their workflow, integrating such tools directly into the framework with a consistent programmatic interface will further improve usability. Moreover, through its interoperability with *spatialdata*, *spatialproteomics* is well positioned to support the combined analysis of spatial proteomics and spatial transcriptomics data within a unified data structure, thereby opening new avenues for multimodal investigation.“

G. References
The references appear appropriate, with all major prior work adequately cited.

H. Clarity and context
The manuscript is well-written and clearly articulated.

Reviewer 3

We thank the reviewer for their feedback on the installation and data availability. Based on this feedback, we have improved both the documentation as well as the presentation of *spatialproteomics* in the manuscript. In particular, we have added example data download links at each relevant section to enable users to follow along with the tutorials.

I encountered an error when attempting to install spatialproteomics alongside other tools such as Cellpose using pip install spatialproteomics[cellpose] on an Apple Silicon Mac. In contrast, the installation proceeded without issues on a Linux (Ubuntu) system.

Thank you for bringing this to our attention. On Mac, it is required to put the package name into quotation marks if it contains brackets (pip install

“spatialproteomics[cellpose]”). We have updated the documentation[13] accordingly. If the error persists, we are happy to further investigate and kindly ask for detailed feedback including a full traceback.

I attempted to test the functionality of spatialproteomics by following the tutorial; however, it was unclear how to obtain the image data and other resources used in the tutorial.To ensure the reproducibility of the tutorial, the authors should clearly specify the source or method for downloading the image data used in the tutorial.

We have updated the notebooks to use data that is accessible with a simple OwnCloud download. This should allow users to easily follow along with the tutorials. In addition, all data associated with the manuscript are deposited at the BioImage Archive in line with the data availability statement.

Other changes to the manuscript

While addressing the reviewer’s comments, we noticed a mistake in the cell type prediction code, which caused the software to predict Stromal cells only based on Podoplanin (and not Podoplanin/CD90, as we showed in the gating tree) and Endothelial cells only based on CD34 (and not CD34/CD31). We have rerun the entire analysis pipeline and adjusted all figures accordingly. None of the initial findings were impacted by this change.

[13] https://sagar87.github.io/spatialproteomics/notebooks/Installation.html

Response to the reviewer

We thank the reviewer for their constructive comments, which helped us to further refine our cell type assignment approach. Based on the suggestions, we have adapted the manuscript to better explain our rationale for using a manual thresholding approach. In addition, we clarified the capabilities and limitations of *spatialproteomics* with respect to multi-platform analyses.

On Question 3, I do appreciate the authors' effort to add a quantitative evaluation of inter-annotator robustness and to compare manual gating with argmax-based labels and astir (Sup. Fig. 4). However, the current response still leaves some ambiguity regarding the impact of threshold choice on the final results. At present, the argument is mainly based on high Cohen's kappa between the three human annotators, but it remains unclear like: (i) how different the actual marker thresholds chosen by each annotator were, and (ii) to what extent reasonable perturbations of these thresholds would affect cell-type proportions or downstream spatial statistics.
In other words, high inter-annotator agreement may partly reflect shared prior expectations and a common gating template, rather than demonstrating that threshold choice has only a minor effect on the outcome.
I would therefore encourage the authors to briefly clarify these points, by reporting the range of thresholds chosen by different annotators for key markers, and adding a simple sensitivity analysis showing how varying selected thresholds within a plausible range affects key quantitative readouts.

We thank the reviewer for their positive assessment of our inter-annotator variability analysis. As we agree that it is important to assess the inter-annotator variability, we provide the thresholds chosen by the different annotators on BioStudies (https://www.ebi.ac.uk/biostudies/bioimages/studies/S-BIAD2100, see tma_thresholds.csv (Annotator 1) and tma_thresholds_from_multiple_annotators.csv (Annotators 2 and 3)). Following the reviewer's suggestion, the figure below shows how the resulting estimates of cell type abundances depend on the thresholds. To dissect this, we varied each threshold for one marker at a time, keeping the other thresholds at their defaults. The nine panels show the resulting estimates of cell type abundance.

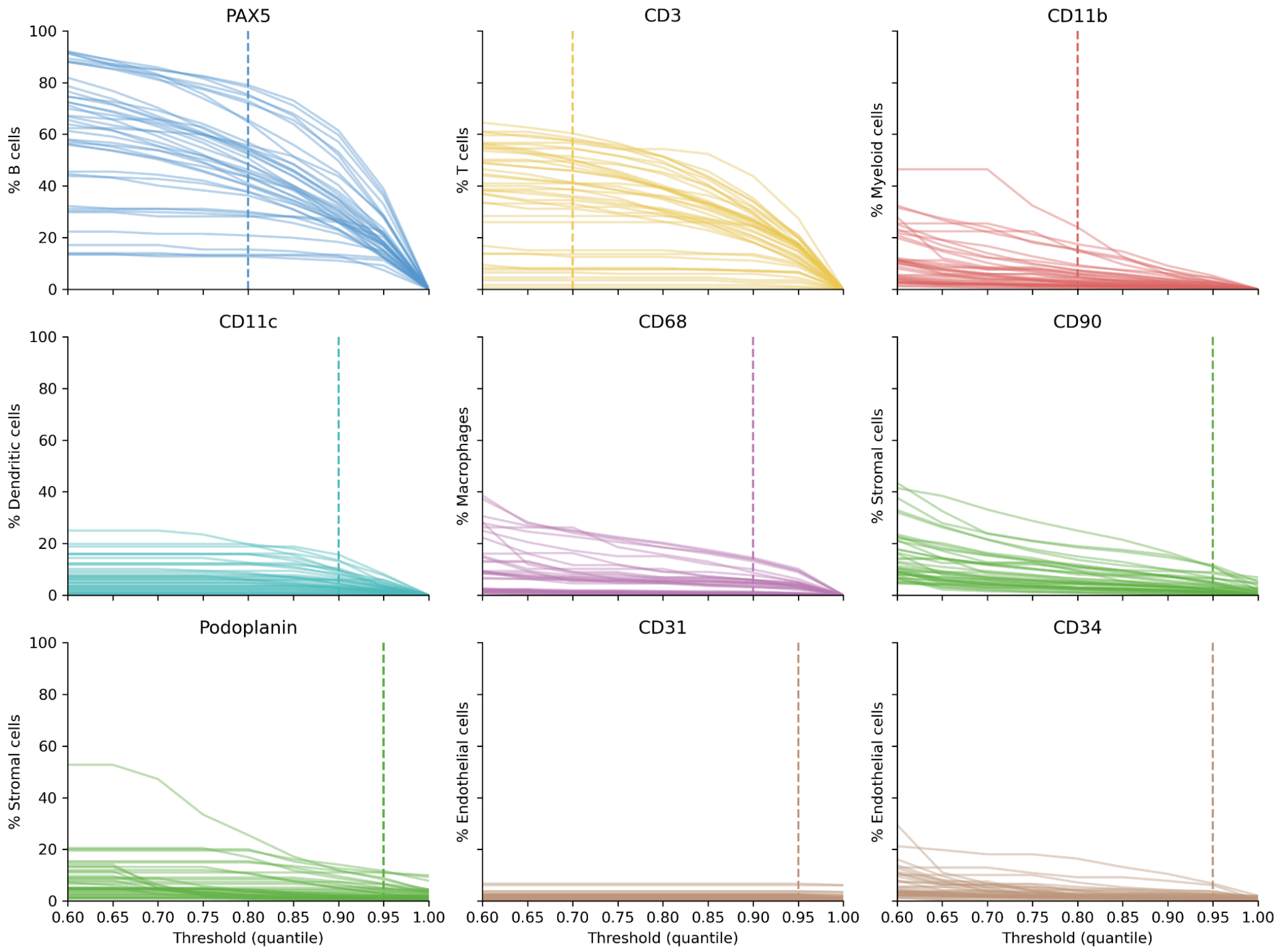

Figure: Relationship between marker thresholds and estimated cell type abundances. For each marker, the default value (selected manually to be appropriate for most samples) is shown by the vertical lines. Multiple lines per panel correspond to multiple samples. Each of the nine panels corresponds to a separate experiment, where the other markers were kept at their default values. The sensitivity of the estimate on threshold choice varies between markers, e.g., it is relatively high for Podoplanin, whereas for CD3, a large range of threshold values around the default lead to essentially unchanged assignments of T cells.

In addition, the response does not fully address the original question about more data-driven thresholding strategies or diagnostic tools. It would be helpful if the authors could explicitly state whether they plan to implement more systematic threshold selection methods or at least provide recommended diagnostic plots in the documentation. Even a concise statement about current limitations and future plans would make the workflow's assumptions more transparent to users.

We agree that automation of cell type assignment, e.g., by automatic setting of these thresholds, is a desirable goal. However, our experience as well as communication by other teams (e.g., https://huggingface.co/spaces/Arozhada/spcelleval) suggest that currently, human-in-the-loop methods still outperform fully automated ones. An important factor is lack of standardisation of the assays, and variabilities in data quality, e.g., due to antibody performance. Given the high value of the data, we therefore currently favour the human-in-the-loop approach. As soon as better methods for automated thresholding emerge, the open and modular design of *spatialproteomics* will make it possible to incorporate these.

We have updated the Discussion as follows.

For the analysis workflow proposed here, we have opted to employ manual thresholding of markers for cell type assignment. While fully automated methods like *astir* [8] exist, they may be sensitive to variable data quality or batch effects, which means in practice that satisfactory results will still require manual intervention, for quality assessment of input data and of output results. Thus, while we anticipate better automation resulting from advances in assay standardization or computational reasoning, the current human-in-the-loop approach is intended to make best use of the data.

On Question 4, the new notebook illustrating the integration of IMC, MICS and CODEX data at the cell-type level is a useful addition. Nevertheless, the response still does not fully address the original concern about cross-platform marker harmonization and multi-batch alignment. As far as I understand, spatialproteomics assumes that each dataset has already been independently normalized and cell-typed, and then operates on a labelled point process, like cell coordinates + cell-type labels for integration.
If this interpretation is correct, I would suggest that the authors make this assumption explicit both in the manuscript and in the documentation: spatialproteomics currently does not provide dedicated functionality for harmonizing partially overlapping marker panels or aligning marker intensities across platforms, and instead relies on users to define comparable cell-type labels upstream. Clarifying this limitation and, if possible, briefly indicating whether marker-level harmonization is a planned future extension would help manage user expectations and make the scope of the toolkit more precise.

We thank the reviewer for highlighting this important point. We have updated the Discussion as follows.

A recurrent study design pattern is the use of multiple spatial profiling platforms, or of the same platform but with different antibody panels, on the same samples. For such studies, we recommend as a relatively broadly applicable and generic approach to perform separate analyses per platform, resulting in cell coordinates, cell type assignments and optionally, further markers of cell state computed from specific protein levels. The resulting labelled point processes can then be compared and integrated using spatial statistics methods [44]. For specific study designs and analytical questions, it may be possible to perform data integration already upstream, e.g., by mapping marker sets into a common latent space [45, 46].

Other comments

For technical correctness, we have replaced the term "interaction" with "spatial association" in the text and figures.

For MCMICRO, the previous table did not contain the full extent of the package. This has now been adjusted accordingly.

**Package**	spatialproteomics	sopa	SPACEc	MCMICRO	SIMPLI	imcRtools/steinbock
**Language**	Python	Python, Snakemake	Python	Nextflow	Nextflow	R, Python
**Data Format**	Xarray or spatialdata	spatialdata	Tif, csv, ...	Tif, csv, ...	Tif, csv, ...	SpatialExperiment
**Enforced Synchronization of Shared Coordinates**	Yes	No	No	No	No	Yes
**Segmentation**	Cellpose, Mesmer, Stardist	Cellpose	Cellpose, Mesmer	Cellpose, Mesmer, Cypository, UnMICST, S3segmenter	CellProfiler, Stardist	Cellpose, Mesmer, CellProfiler
**Image Processing**	Fully customizable	None	None	Background correction	Thresholding	Hot Pixel Removal
**Protein Quantification**	Fully customizable	Mean, min, max	Mean	MCQuant	CellProfiler	Mean
**Expression Matrix Processing**	Z-score, Double Z-score, MinMax, ArcSin, Clip	Z-score, Spillover Correction	Z-score, Double Z-score, MinMax, ArcSin	Log1p, Rescale, ComBat	CellProfiler	Spillover Compensation
**Cell Phenotyping**	Astir, Gating (Argmax, Binarization)	Argmax	SVM, STELLAR, Clustering	Clustering, Gating	Clustering, Thresholding	Random Forest, Clustering
**Neighborhood Analysis**	Clustering	NA	Clustering	SCIMAP (Spatial LDA, Spatial Lag)	Clustering	Clustering, lisaClust
**Interactivity**	Napari	Napari	TissUUmaps	Napari (CyLinter), Minerva	NA	Napari (napari-imc), cytoviewer/cytomapper

Table 1: Comparison of different tools for analyzing highly multiplexed immunofluorescence images.

1. The documentation of spatialproteomics is clear and well-structured, with tested code examples and easy-to-follow tutorials. The guides on image processing and cell type annotation are especially user-friendly. However, key functions like neighborhood analysis and integration with downstream tools (e.g., squidpy in Python or spatstat in R) are either shown only in separate examples or not covered in detail. Including these features in a unified workflow would improve usability, especially for new users unfamiliar with spatial analysis or the broader scverse ecosystem.

2. The authors provide a dedicated section on interoperability, with support for exporting to Zarr, CSV, AnnData, and SpatialData, making it easy to integrate with Python tools like scanpy and squidpy. However, there is no mention of support for R-based frameworks such as Seurat, which are still widely used in spatial transcriptomics. Although spatstat in R is used for spatial statistics like Ripley's K, it's unclear whether this is part of an integrated workflow or run separately. Including an example that bridges into R, or clarifying plans for R compatibility, would make the toolkit more versatile.

3. The current marker thresholding strategy for cell type classification relies on manual tuning, starting from fixed quantiles and adjusted by visual inspection. While this offers flexibility, it introduces subjectivity and may limit reproducibility. It would be helpful if the authors could clarify whether they plan to include more data-driven thresholding methods (e.g., adaptive thresholding or mixture models), or at least provide heuristics or diagnostic plots to guide users. This would reduce the need for trial-and-error, especially for users with limited prior knowledge. Additionally, since the annotation is based on decision trees with quantile thresholds, some form of benchmarking—such as consistency metrics, F1 scores, or agreement with expert labels—would help justify threshold choices and improve transparency.

4. The current version of spatialproteomics seems best suited for single-batch datasets with consistent marker panels, as demonstrated in the BNHL use case. However, it is unclear how the package handles cross-sample integration when marker sets differ, such as in multi-batch designs or across platform versions. Even if not currently supported, it would be useful for the authors to clarify whether marker harmonization or multi-sample alignment is a planned feature. Furthermore, demonstrating the toolkit's ability to integrate or compare datasets from different imaging modalities (e.g., CODEX, IMC, MIBI), which often share only partial marker overlap and differ in data characteristics, would strengthen its value. Clear guidance or examples on how such cross-platform analyses could be approached using spatialproteomics would significantly broaden its applicability.

5. While the authors highlight the use of “xarray” and “dask” to support scalable data handling, the manuscript lacks a systematic evaluation of performance in terms of runtime, memory usage, and hardware requirements. It would be valuable to include concrete metrics—such as peak memory, processing time, and CPU/GPU specs—especially for full whole-slide images or large TMA cohorts. This information would help guide users with limited computing resources. Additionally, beyond the session details in the documentation, a more thorough benchmarking analysis showing how performance scales with image size, number of channels, or cell count would strengthen the claims of scalability and robustness, and make the toolkit more practical for broader use cases.

6. The package usefully integrates several leading tools for segmentation and spatial analysis (e.g., cellpose, stardist, mesmer, squidpy) through a consistent and user-friendly interface, which is a major strength. However, given the rapid development of new methods in this field, it would be helpful if the authors could clarify how easily users can incorporate alternative tools. For instance, if a new segmentation model is developed, can users load custom segmentation masks or label images into the spatialproteomics object for downstream analysis? Likewise, can existing neighborhood graphs or clustering steps be replaced with user-defined logic, such as GNN-based representations? Even if full modularity is not intended, guidance on how to connect external outputs to the pipeline—via format compatibility or accessor injection—would support broader adoption and adaptability in evolving workflows.
